# The first complete chloroplast genome of *Thalictrum fargesii*: insights into phylogeny and species identification

**DOI:** 10.3389/fpls.2024.1356912

**Published:** 2024-04-29

**Authors:** Shixi Chen, Fardous Mohammad Safiul Azam, Mst. Lovely Akter, Li Ao, Yuanchao Zou, Ye Qian

**Affiliations:** ^1^ College of Life Science, Neijiang Normal University, Neijiang, Sichuan, China; ^2^ Fishes Conservation and Utilization in the Upper Reaches of the Yangtze River, Key Laboratory of Sichuan Province, Neijiang Normal University, Sichuan, China; ^3^ Department of Biotechnology and Genetic Engineering, Faculty of Life Sciences, University of Development Alternative, Dhaka, Bangladesh; ^4^ Key Laboratory of Regional Characteristic Agricultural Resources, College of Life Sciences, Neijiang Normal University, Neijiang, Sichuan, China; ^5^ Branch of The First Affiliated Hospital of Xinjiang Medical University, Changji, Xinjiang, China

**Keywords:** chloroplast genome, *Thalictrum fargesii*, thalidasine, repeat sequences, RSCU, nucleotide diversity, variant sites, phylogeny

## Abstract

**Introduction:**

*Thalictrum fargesii* is a medicinal plant belonging to the genus *Thalictrum* of the Ranunculaceae family and has been used in herbal medicine in the Himalayan regions of China and India. This species is taxonomically challenging because of its morphological similarities to other species within the genus. Thus, herbal drugs from this species are frequently adulterated, substituted, or mixed with other species, thereby endangering consumer safety.

**Methods:**

The present study aimed to sequence and assemble the entire chloroplast (cp) genome of *T. fargesii* using the Illumina HiSeq 2500 platform to better understand the genomic architecture, gene composition, and phylogenetic relationships within the Thalictrum.

**Results and discussion:**

The cp genome was 155,929 bp long and contained large single-copy (85,395 bp) and small single-copy (17,576 bp) regions that were segregated by a pair of inverted repeat regions (26,479 bp) to form a quadripartite structure. The cp genome contains 133 genes, including 88 protein-coding genes (PCGs), 37 tRNA genes, and 8 rRNA genes. Additionally, this genome contains 64 codons that encode 20 amino acids, the most preferred of which are alanine and leucine. We identified 68 SSRs, 27 long repeats, and 242 high-confidence C-to-U RNA-editing sites in the cp genome. Moreover, we discovered seven divergent hotspot regions in the cp genome of *T. fargesii*, among which *ndhD-psaC* and rpl16-rps3 may be useful for developing molecular markers for identifying ethnodrug species and their contaminants. A comparative study with eight other species in the genus revealed that *pafI* and *rps19* had highly variable sites in the cp genome of *T. fargesii*. Additionally, two special features, (i) the shortest length of the *ycf1* gene at the IRA-SSC boundary and (ii) the distance between the *rps19* fragment and *trnH* at the IRA-LSC junction, distinguish the cp genome of *T. fargesii* from those of other species within the genus. Furthermore, phylogenetic analysis revealed that *T. fargesii* was closely related to *T. tenue* and *T. petaloidium*.

**Conclusion:**

Considering all these lines of evidence, our findings offer crucial molecular and evolutionary information that could play a significant role in further species identification, evolution, and phylogenetic studies on *T. fargesii*.

## 1 Introduction

The genus *Thalictrum* belongs to the subfamily Thalictroideae of Ranunculaceae, with the subgenera *Thalictrum* and *Lecoyerium*, which includes approximately 200 species and is distributed in Asia, Europe, Africa, North America, and South America (Zhu and Xiao, 1989). At least 43 species of this genus have been used medicinally (Chen et al., 2003), 26 of which have been used as medicinal herbs in China and have been found to contain bioactive compounds, such as thalidomide, thalicarpine, lithospermoside, and dasycarponin (Wu et al., 1979; Huang and Williams, 1998). Among them, *Thalictrum fargesii* Franch. ex Finet & Gagnep. is distributed throughout Asia and has been reported to have ethnomedicinal and traditional uses in the Tibetan region of China (Zhang et al., 2021) and the Himalayan region of India (Singh et al., 2016). In China, *T. fargesii* is geographically distributed in the Anhui, Chongqing, Fujian, Gansu, Guizhou, Henan, Hubei, Hunan, Jiangxi, Shaanxi, Sichuan, and Zhejiang Provinces (Wang and Wang, 1979; Fu and Zhu, 2001; Xie, 2016; Wang et al., 2018). Thalictroideae is a monophyletic group that is well supported by systematic molecular studies of Ranunculaceae (Johansson and Jansen, 1993; Hoot, 1995; Johansson, 1995; Ro et al., 1997; Wang and Chen, 2007). Ren et al. (2011) first examined detailed floral development in this subfamily. They classified this species based on chloroplast *rbcL*, *matK*, *trnL-F*, and nuclear ITS primers (Wang and Chen, 2007), and a taxonomic report on this species was provided by Zeng et al. (2021).

The plant of *T. fargesii* is termed “CHENG KOU TANG SONG CAO” in China for its thalidasine roots (Zhou et al., 2011). The roots of this species are also rich in the naturally active compounds 5-methoxy salicylic acid, thalfoetidine, thaligasinine, and thalisopidine (Wu and Yi, 1991; NCBI, 2022). Alkaloids from this species have been reported to exhibit various pharmacological activities including antitumor, antimicrobial, antitussive, antiamoebic, antiparasitic, antiplatelet aggregation, antisilicosis, hypotensive, and HIV antiviral activities (Gao, 1999; Singh et al., 2023). This species is used in traditional Chinese medicine to treat dysentery, diarrhea, viral hepatitis, influenza, measles, carbuncles, boils, and eye congestion (Chen et al., 2003). A pharmacophylogenic study showed that aporphine, protopine, pavine, phenanthrene, and bisbenzylisoquinoline are prominent in the *Thalictrum* genus (Da-Cheng et al., 2015). Additionally, metabolites related to their structural features and roles have been studied in *T. fargesii* (Khamidullina et al., 2006).

Because of its immense potential in herbal medicine, this species is collected from the wild or grown by rural farmers in China. Consequently, in local herbal markets, the species is often confused by morphological similarities with other related species in the genus, and adulterated, altered, or mixed in the herbal formulation with or without intention (Bainsal et al., 2022). No doubt, it poses a serious threat to consumer safety. Thus, proper identification of *T. fargesii* is of great importance in herbal formulations, which are often misunderstood, confused, or incorrectly recorded during plant identification. For example, during the course of herbarium specimens, *T. fortune* was found to be mixed with *T. fargesii* Franchet ex Finet & Gagnepain (1903: 608), resulting in wrong records of the geographical distribution of these species (Zeng et al., 2022). Moreover, Li et al. (2016) clarified the variations in *T. fargesii* and proposed a synonym for *T. xingshanicum*, revealing that the name of *T. fargesii* was previously misunderstood. In addition, herbarium collections of *T. fargesii* from Neijiang, Sichuan Province were misled by misidentification of the collections of *T. ramosum* in Gansu Province (Zeng et al., 2023). These misidentifications can be explained by the morphological variation related to environmental factors (e.g., weather, soil properties, elevation level, etc.) where the plants have grown (Hernández-Nicolás et al., 2017). It is important to eliminate such dilemmas using molecular approaches rather than morphological observations (Duminil and Di Michele, 2009).

Identifying member species within the Ranunculaceae family, especially within the *Thalictrum*, is taxonomically challenging because of the inability to distinguish these species through universal molecular markers and similar morphological traits (Li et al., 2020). Previous studies have revealed that *Thalictrum* species form a highly supported clade, indicating that they are monophyletic (Cai et al., 2022; Michimoto et al., 2022). *Thalictrum* species exhibit small chromosomes compared with those of Rununculus, and this genus is challenging and diverse in terms of taxonomy and phylogeny based on variations in morphological traits (Langlet, 1927; Tamura, 1995; Xiang et al., 2022). As a critical solution, chloroplast (cp) genomic resources have facilitated the classification of members of the Ranunculaceae family, providing data and insight into evolutionary relationships (Zhai et al., 2019), phylogenetic resolution (bootstrap support and tree certainty) (Morales-Briones et al., 2019), and marker exploration (Xiang et al., 2022).

In the photosynthesis of green plants, chloroplasts play the most significant role in coordinating the expression of different genes between these organelles and the nuclear genome in response to environmental stress, including cellular signaling (Daniell et al., 2016; Serrano et al., 2016; Yu et al., 2019; Tano et al., 2023). Interestingly, because of the presence of inherited conserved genes and their organization and low recombination and substitution rates, compared with nuclear genomes, cp genomes serve as resources for phylogenetic analysis and evolutionary studies (Wolfe et al., 1987; Corriveau and Coleman, 1988; Birky, 2001; Provan et al., 2001; Zhang et al., 2003; Ravi et al., 2008). Moreover, DNA barcoding and molecular breeding have been studied using cp genomes (Daniell et al., 2016). Several strategies have been adopted to sequence the organelle genome, including amplification of the whole cp genome using a universal set of primers, sequencing (Cronn et al., 2008; Dong et al., 2013), and using total genomic DNA to extract the cp genome (Velasco et al., 2007; McPherson et al., 2013). Considering the available taxonomic evidence, the importance of cp genome studies of folk medicinal plants has been demonstrated by several studies (He et al., 2021; Lin et al., 2021; Pu et al., 2022).

From several previous studies, significant molecular cross-talks have been reported on the characteristics of cp genomes within *Thalictrum*, such as *T. cirrhosum* (Zhe et al., 2023)*, T. coreanum* (Park et al., 2015), *T. foliolosum* (Pu et al., 2022), and *Thalictrum aquilegiifolium* var. *sibiricum* (Michimoto et al., 2022). Current research has focused predominantly on *T. fargesii*, emphasizing the need to construct the whole cp genome using next-generation sequencing, characterization, long and simple sequence repeats (SSRs), phylogenetic analysis, nucleotide diversity, and selection pressure of genes within the *Thalictrum*. This study aimed to generate molecular data for the cp genome of *T. fargesii* to provide taxonomic and molecular information to biologists, practitioners, and professionals in herbal medicine to develop molecular markers to avoid misidentification.

## 2 Materials and methods

### 2.1 DNA extraction and next-generation sequencing

The *T. fargesii* plant sample used for cp genome sequencing was identified and artificially reproduced in the Wenchuan Botanical Garden of the Aba Tibetan and Qiang Autonomous Prefecture in Sichuan Province, China. The voucher specimen (Accession No. CP00002) was identified and deposited at the Herbarium of Neijiang Normal University (Neijiang City, China; Shixi Chen, saihei@foxmail.com). After collecting leaf specimens, they were stored at room temperature and packaged in 0.2 g of silicon dioxide. We used a modified cetyltrimethylammonium bromide (CTAB) method to extract total genomic DNA from fresh leaves (Porebski et al., 1997). Short reads of the *T. fargesii* cp genome were sequenced using a genomic library with an insert size of 260 bp. The products were prepared and sequenced on an Illumina HiSeq 2500 platform with approximately 436× coverage.

### 2.2 Chloroplast genome assembly and annotation

The sequenced reads were filtered using the Trimmomatic program (version 0.39) (Bolger et al., 2014). The filtered reads were assembled into the cp genome using NOVOPlasty (version 4.3.1) (Dierckxsens et al., 2017), and annotation was conducted using GeSeq with 3rd Party Stand-Alone Annotators from Chloë (version 0.1.0) and tRNA annotation from tRNAscan-SE (version 2.0.7) (Tillich et al., 2017). The annotation was followed by a manual check against the information from NCBI. Later, the sequences were deposited in the NCBI database (GenBank accession No. ON868919.1). The *T. fargesii* cp genome was visualized and plotted using Chloroplot (web-based tool: https://irscope.shinyapps.io/Chloroplot/) (Zheng et al., 2020).

### 2.3 Comparison of the chloroplast genome within Thalictrum

Eight relevant *Thalictrum* cp genome sequences were downloaded from the GenBank database. Nine sequences, including *T. fargesii*, were analyzed to determine the degree of variation and sequence conservation within the same genus (**Supplementary Table 1**). We compared the IR regions of all species using the web-based tool IRplus (https://irscope.shinyapps.io/IRplus/) to visualize the IR-SC boundaries and their gene orientations (Díez Menéndez et al., 2023). Sequence alignment was performed to identify the variation in sites between the nine *Thalictrum* cp genome sequences on mVISTA using the alignment program LAGAN (Mayor et al., 2000; Frazer et al., 2004). The sites were checked manually using BioEdit version 7 (Hall, 1999). The sites were subsequently analyzed, and the results are presented in **Supplementary Table 2**.

### 2.4 Relative synonymous codon usage analysis

Each amino acid has a minimum of one codon and a maximum of six codons owing to the simplicity of the codons. MEGA11 software was used to calculate the relative synonymous codon usage (RSCU) in the cpDNA of *T. fargesii* (Tamura et al., 2021).

### 2.5 Repeat analysis

SSR locus analysis was performed on the assembled *T. fargesii* cp genome sequence using the MicroSarellite identification tool (MISA) (Beier et al., 2017). The parameters were set as follows: 1–10, 2–5, 3–4, 4–3, 5–3, and 6–3, and the minimum distance between the two SSRs was set to 0 bp. The software package REPuter was used to conduct a long-repeat analysis and a minimum repeat size of 30 bp was assigned along a Hamming distance of 3 bp (Kurtz et al., 2001). The tandem repeat finder is an online program that detects tandem repeats using the default parameter settings (Benson, 1999).

### 2.6 Predicting RNA editing sites

We predicted RNA editing sites in the cp genome of *T. fargesii* using a convolutional neural network (CNN) model-based tool called Deepred-mt (Edera et al., 2021). We extracted the PCGs of this cp genome and inputted them into the Deepred-mt tool for prediction, considering threshold probability values greater than 0.9 as reliable results.

### 2.7 Synonymous substitution and selective pressure

The natural selection that drives molecular evolution was analyzed for the *Thalictrum* chloroplast genome; thus, we calculated the ratio of nonsynonymous (dn) to synonymous (ds) substitutions called ω (dn/ds). The ω value is an indicator of natural selection of PCGs. Values ω > 1, ω = 1, and ω < 1 indicate positive, neutral, and negative/purifying selection, respectively. The stop codons of all 73 PCGs from these species were deleted and subsequently aligned, and the dn, ds, and ω values were calculated using MEGA 11 (Tamura et al., 2021).

### 2.8 Phylogenetic analysis

To ascertain the phylogenetic position of *T. fargesii* within *Thalictrum*, nine species were analyzed, and *Aconitum delavayi* (NC_038097) was chosen as the outgroup. Sequence (full-length) alignment was performed using the MAFFT v7 plugin integrated into PhyloSuite v1.1.15 (Katoh and Standley, 2013). The dataset was refined using the Gblocks program with stringent parameter settings and then subjected to maximum likelihood (ML) analyses. Substitutional saturation was assessed using DAMBE version 7.0.68 (Xia, 2018) and phylogenetic trees were constructed using ML algorithms. ML phylogenies were conducted using RAxML v8.2.12, with 1,000 bootstrap replicates, and the GTRGAMMA model (Stamatakis, 2014).

## 3 Results

### 3.1 Features of the chloroplast genome

**Figure 1:**
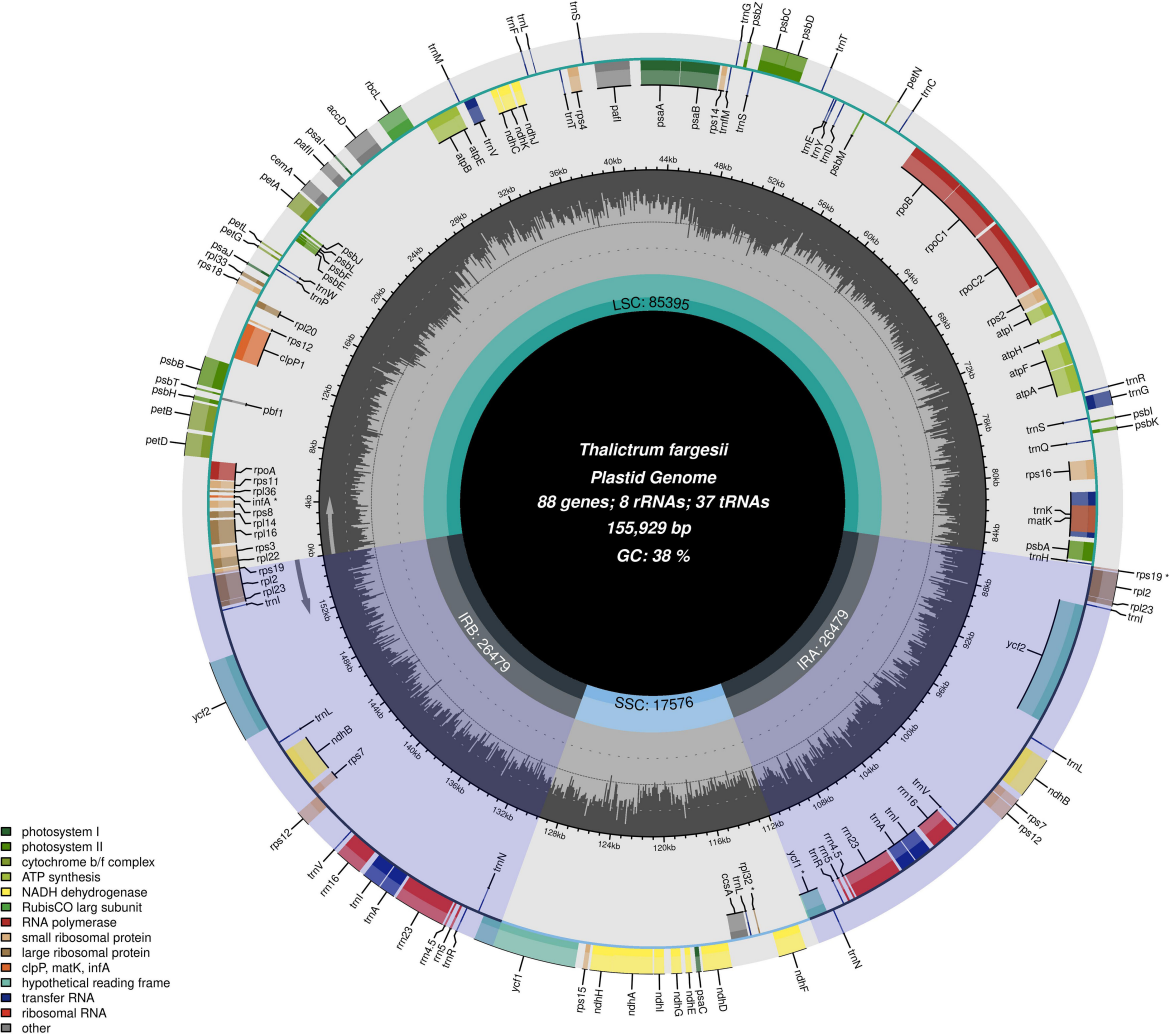
Graphical representation of the features identified in the cp genome of *T. fargesii*. The species name, genome length, GC content, and number of genes are represented in the center of the plot. Represented by arrows, transcripts for genes inside and outside the circle are generated in opposite directions of the tetrad structure of the cp genome. Different colors are used to distinguish between genes that belong to specific functional categories. At the lower left, the legend for the classification of the cp genes according to their function is shown. The darker shaded region inside the inner circle indicates the GC content, while the light color indicates the AT content of the cp genome. The gradient GC content of the cp genome is represented by the second circle, with a zero level based on the outer circle. The GC content of each gene is displayed as the proportion of shaded areas. The boundaries of the small single-copy (SSC), large single-copy (LSC), and inverted repeat (IRA, IRB) regions are denoted in the inner circle.

After removing the adapter and low-quality reads, we found 9.4 Gb data for the *T. fargesii* cp genome. The cp genome was 155,929 bp in length, displayed a circular structure, and was divided into typical quadripartite regions, similar to most land plants (**Figure 1**). The cp genome includes two 26,479-bp inverted repeats (IRA and IRB), and they separated the genome into a large single-copy (LSC) region of 85,395 bp and a small single-copy (SSC) region of 17,576 bp (**Supplementary Table 1**). The overall GC content of the cp genome was 38%.

The size of the cp genomes of the *Thalictrum* ranged from 154,889 bp (*T. thalictroides*) to 156,103 bp (*T. tenue*). The cp genome of *T. fargesii* (155,929 bp) was the fourth largest and most similar to those of other species within the genus (**Supplementary Table 1**). Moreover, we found the most extended LSC region (85,395 bp) in the *T. fargesii* cp genome, and the shortest LSC region (84,733 bp) in *T. coreanum*. In terms of count, the total number of genes found ranged from 128 to 134, and for PCGs, it ranged from 83 to 89. Nonetheless, except for *T. viscosum* (38 tRNAs), most species, including *T. fargesii*, possess 37 tRNA genes.

### 3.2 Gene composition of the chloroplast genome

Annotation of the cp genome revealed 133 genes, including 88 PCGs (including six duplicated genes), 37 tRNA genes (including seven duplicated genes), and 8 ribosomal RNA genes. The identified genes were categorized into four groups: photosynthesis-related genes (*n* = 45), self-replicating genes (*n* = 58), other genes (*n* = 6), and unknown genes (*n* = 6) (**Table 1**). Four pseudogenes have been identified in the genome. The IR regions carried two copies of *ndhB, rps7, rps12, rpl2, rpl23, ycf2, trnI-CAU, trnL-CAA, trnV-GAC, trnI-GAU, trnA-UGC, trnR-ACG, trnN-GUU, rrn16, rrn23, rrn4.5*, and *rrn5*. Of the genes identified, 21 contained introns and 16 (*trnK-UUU, trnG-UCC, petB, petD, rpl16, rpl2, ndhB, rps12, trnI-GAU, trnA-UGC, rrn23, trnA-UGC, trnI-GAU, rps12, ndhB*, and *rpl2*) contained one intron. The other five genes (two copies of *rps16, trnV-UAC*, and *atpF*) contained two introns (**Supplementary Table 3**). Moreover, 10 genes were located in the LSC region, 10 in the two IR regions, and 1 in the SSC region. The largest intron (2,532 bp) was observed in *trnK-UUU*, and the smallest intron (199 bp) was found in *rrn23*. tRNA genes are distributed throughout the genome and are encoded by 61 possible codons (excluding the stop codon).

**Table 1:** Genes according to the categories within the *T. fargesii* chloroplast genome.

Category	Gene group	Gene name*
Photosynthesis	Subunits of photosystem I	*pafI, pafII, psaA, psaB, psaC, psaI, psaJ*
	Subunits of photosystem II	*psbA, psbB, psbC, psbD, psbE, psbF, psbH, psbI, psbJ, psbK, psbL, psbM, psbT, psbZ*
	Subunits of NADH dehydrogenase	*ndhA, ndhB^di^, ndhC, ndhD, ndhE, ndhF, ndhG, ndhH, ndhI, ndhJ, ndhK*
	Subunits of cytochrome b/f complex	*petA, petB^i^, petD^i^, petG, petL, petN*
	Subunits of ATP synthase	*atpA, atpB, atpE^e^, atpF, atpH, atpI*
	Large subunit of rubisco	*rbcL*
Self-replication	Proteins of large ribosomal subunit	*rpl14, rpl16^i^, rpl2^di^, rpl20, rpl22, rpl23^d^, rpl33, rpl36*
	Proteins of small ribosomal subunit	*rps11, rps12^di^, rps14, rps15, rps16^e^, rps18, rps19, rps2, rps3, rps4, rps7^d^, rps8*
	Subunits of RNA polymerase	*rpoA, rpoB, rpoC1, rpoC2*
	Ribosomal RNAs	*rrn16^d^, rrn23^d^, rrn4.5^d^, rrn5^d^*
	Transfer RNAs	*trnA-UGC^di^, trnC-GCA, trnD-GUC, trnE-UUC, trnF-GAA, trnG-GCC, trnG-UCC^i^, trnH-GUG, trnI-CAU^d^, trnI-GAU^di^, trnK-UUU^i^, trnL-CAA^d^, trnL-UAA, trnL-UAG, trnM-CAU, trnN-GUU^d^, trnP-UGG, trnQ-UUG, trnR-ACG^d^, trnR-UCU, trnS-GCU, trnS-GGA, trnS-UGA, trnT-GGU, trnT-UGU, trnV-GAC^d^, trnV-UAC^e^, trnW-CCA, trnY-GUA, trnfM-CAU*
Other genes	Acetyl-CoA carboxylase	*accD*
	Megakaryocyte-associated tyrosine kinase	*matK*
	ATP-dependent CLP protease	*clpP1*
	N-terminal nucleophile amino hydrolase superfamily	*pbf1*
	Envelope membrane protein	*cemA*
	c-type cytochrome synthesis gene	*ccsA*
Genes of unknown function	Conserved hypothetical chloroplast ORF	*ycf1, ycf2^d^*
	Pseudogene	*rpl32, rps19-*fragment*, ycf1, infA*

*d, duplicate gene; i, genes with one intron; e, genes with two introns.

As shown in **Table 1**, the PCG genes have different functions, including one acetyl-CoA carboxylase, six ATP synthases, one ATP-dependent CLP protease, five cytochrome b6, one cytochrome c biogenesis protein, one cytochrome b/f, one envelope membrane protein, two hypothetical chloroplast RF2, one megakaryocyte-associated tyrosine kinase, 12 NADH dehydrogenases, one N-terminal nucleophile amino hydrolase superfamily, seven photosystem I, 14 photosystem II, 25 ribosomal proteins, one ribulose-1,5-bisphosphate carboxylase/oxygenase large subunit, and four RNA polymerases. Each RNA had two copies and was equally distributed within the IR region. Duplicated tRNAs were not present in the LSC region. Moreover, *rpl32*, the *rps19* fragment, one copy of *ycf1*, and *infA* were pseudogenes.

### 3.3 The base composition of the chloroplast genome

Base compositions of the LSC, SSC, and IR regions and codons at various locations were examined in the cp genome. The percentages of GC content in LSC, SSC, and IRs were 40.57%, 32.53%, and 43.23%, respectively, indicating that the percentage in the IR region was greater than that in the other regions. Moreover, the frequencies of adenine (A), thymine (T), guanine (G), and cytosine (C) contents were 30.46%, 31.12%, 18.84%, and 19.58%, respectively. The single-copy regions, LSC (59.43%) and SSC (67.48), had higher AT content than the repeat regions (56.77%) (**Table 2**). The reduced AT content in the IR regions may be related to the presence of rRNA, which contains fewer AT nucleotides, contributing to genome stability and sequence complexity. Similarly, the CSD (protein-coding region) was rich in AT (61.24%), whereas the GC content was 38.73%. At the first, second, and third codon positions, each had a length of 25,038 bp and contained a range of 29.10%–31.44% A, 23.44%–37.25% T, 16.69%–27.35% G, and 14.61%–20.67% C contents, respectively. These findings disclosed that the cp genome had a higher AT than the GC content and that the codons preferred bases A and T.

**Table 2:** Nucleotide composition in different regions of the chloroplast genome of *T. fargesii*.

Region	Size (bp)	A (%)	T (%)	G (%)	C (%)	GC (%)
Total genome	155,929	30.46	31.12	18.84	19.58	38.42
LSC	85,395	29.76	29.67	20.06	20.51	40.57
SSC	17,576	33.93	33.55	15.35	17.18	32.53
IRA	26,479	28.21	28.56	20.97	22.26	43.23
IRB	26,479	28.56	28.21	22.26	22.97	43.23
CDS	75,114	30.28	30.96	20.70	18.06	38.76
1st codon position	25,038	30.31	23.44	27.35	18.90	46.25
2nd codon position	25,038	29.10	32.18	18.06	20.67	38.73
3rd codon position	25,038	31.44	37.25	16.69	14.61	31.30

### 3.4 Codon usage

**Figure 2:**
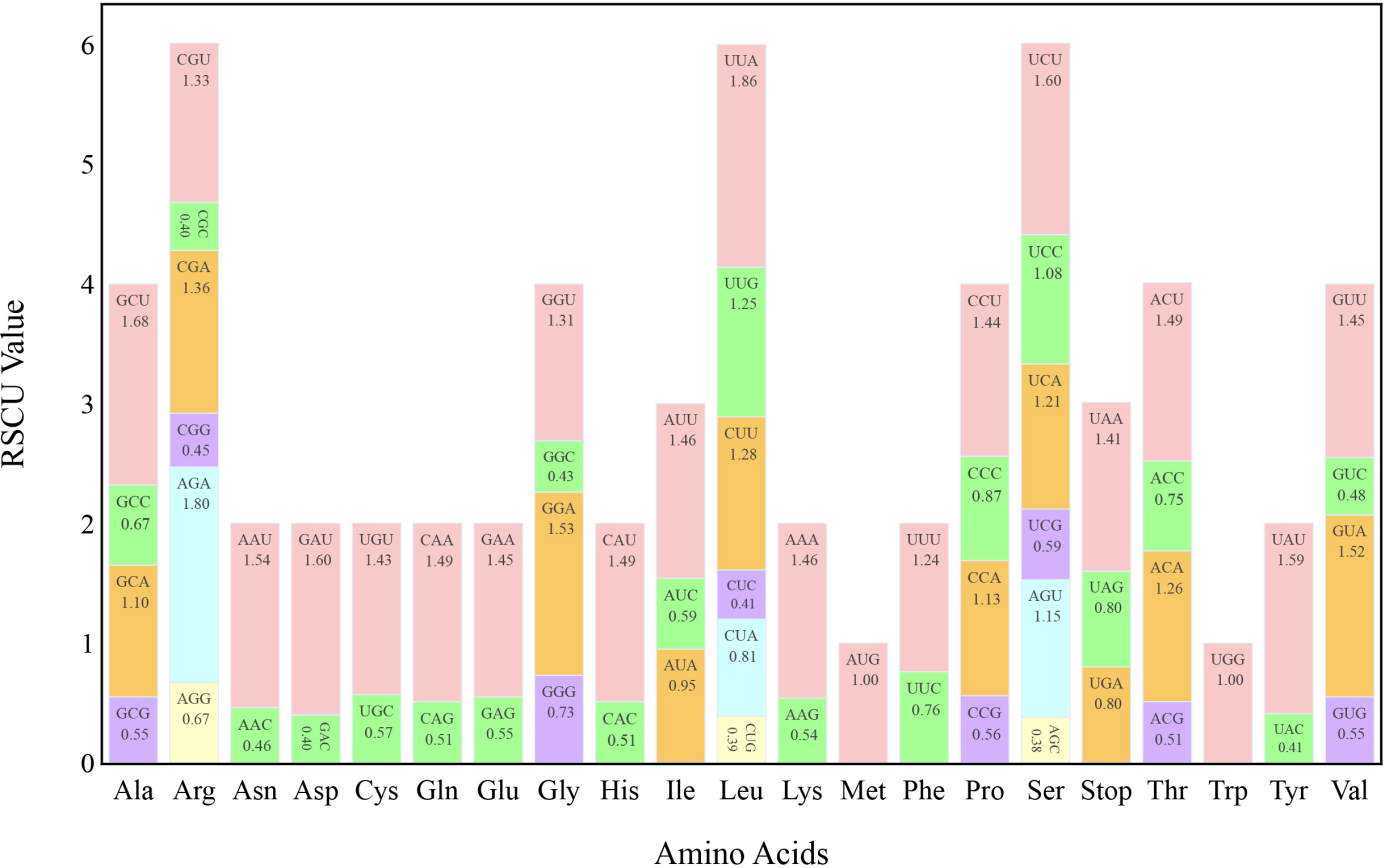
Relative synonymous codon usage (RSCU) of 20 amino acids and stop codons in all protein-coding genes of the chloroplast genome of *T. fargesii.* The colors of the histograms correspond to the colors of the codons.

The ratio of the frequency of a codon observed to that expected, termed “relative synonymous codon usage” (RSCU), was determined, which indicates codon usage bias in coding sequences. The protein-coding sequences (CDSs) of the *T. fargesii* cp genome were used to calculate RSCU via MEGA 11, and 64 codons were found. Three of these were stop codons and 61 encoded 20 amino acids. A single codon encodes methionine (Met) and tryptophan (Try), whereas the other amino acids are encoded by two to six codons (**Figure 2**). Among the 64 codons, 31 had RSCU > 1 in the CDS of the cp genome, indicating that they were high-frequency and optimal codons. A or U was preferred as the third base of the high-frequency codon, as evidenced by the fact that 16 of these codons ended in U, 13 in A, 1 in G, and 1 in C. The most preferred codon was GCU, which encodes alanine (Ala) and has an RSCU value of 2. UUA, which encodes leucine (Leu) and has an RSCU value of 1.86, was the second-most preferred codon.

### 3.5 Simple sequence repeats

**Figure 3:**
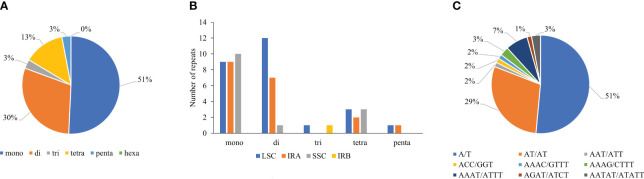
Repeat type and number of analyzed SSRs in the cp genome of *T. fargesii*. **(A)** SSR-type distribution and their frequency in the complete cp genome. **(B)** Distribution of SSRs in the LSC, SSC, and IR regions. **(C)** Type and frequency of each identified SSR.

SSRs from cp genomes can be used to explore evolutionary relationships and population genetics, owing to their high polymorphism rates and consistent repetition (Xia et al., 2022). Most SSRs are A or T units that contribute to the AT richness of the chloroplast genome (Chew et al., 2023). Analysis of SSRs using the MISA web-based tool (Beier et al., 2017) revealed that the *T. fargesii* cp genome comprises 68 SSR loci. The most abundant were mononucleotides (35, 51%), followed by dinucleotides (20, 30%), trinucleotides (2, 3%), tetranucleotides (9, 13%), and pentanucleotides (2, 3%) (**Supplementary Table 4**) (**Figure 3A**). Hexanucleotide repeats were absent. Among these repeats, 43.33%, 23.33%, and 25% were present in the LSC, SSC, and IR region, respectively (**Figure 3B**). In the cp genome of *T. fargesii*, the single-base repeat unit A/T (51%) is the most prevalent, followed by AT/TA (29%), AAAT/ATTT (7%), AATAT/ATATT (3%), and AAT/ATT (2%). The forenamed repeat units accounted for 92% of the total SSRs, whereas G/C repeat units accounted for only 8% of the total SSRs (**Figure 3C**). Among the SSRs, 21 were located in the intergenic region, 45 were within the CDS region, and the remaining 2 were miscellaneous.

### 3.6 Long repeats

**Figure 4:**
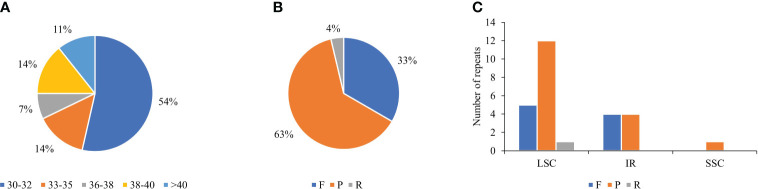
Distribution types and number of long repeats in the cp genome of *T. fargesii*. **(A)** Frequencies of different groups of repeats according to their length in the cp genome. Different colors indicate repeats of various sizes in bp. **(B)** Frequencies of different types of repeats: forward **(F)**, palindrome (P), and reverse (R). **(C)** Distribution of the long repeats in the LSC, SSC, and IR regions. Different colors indicate the types of repeats.

Long repetitive sequences have proven to be valuable markers for comparative genomics, phylogenetics, genomic recombination studies, and plant evolution (Ivanova et al., 2017). According to our analysis, *T. fargesii* consisted of major repetitive sequences between 30 and 40 bp in length, including 30–32 bp, 33–35 bp, 36–38 bp, 38–40 bp, and ≥40 bp, accounting for 15, 4, 2, 4, and 3 repeats, respectively (**Figure 4A**). In total, the cp genome contained 27 long repeats, namely, 17 palindromic repeats (P), 9 forward repeats (F), 1 reverse repeat (R), and no complementary repeats (**Supplementary Table 5**) (**Figure 4B**). Among the total repeats, 66.66% were in the LSC region, 29.62% were in the IR region, and only 3.70% were in the SSC region (**Figure 4C**). Only six genes (*pafI*, *psaB*, *trnS-GCU*, *rpl16*, *ccsA*, and *ycf2*) possessed long repeat elements, and *ycf2* contained the highest number of repeat sites (*n* = 8, 29.62%). The remaining repeats were located in intergenic regions.

### 3.7 Tandem repeat analysis

Centromeres are essential for chromosomal segregation and contain megabase-scale arrays of tandem repeats. These tandem repeats share common properties among species across different phyla (Melters et al., 2013). In total, 21 tandem repeats were identified in the cp genome of *T. fargesii*, 12 of which were dispersed in the gene spacer regions (*rpoB/trnC-GCA*, *petN/psbM*, *petN/psbM*, *petN/psbM*, *psbZ/trnG-GCC*, *psbZ/trnG-GCC*, *atpB/rbcL*, *trnW-CCA/trnP-UGG*, *trnP-UGG/psaJ*, *trnP-UGG/psaJ*, *rps12/trnV-GAC*, and *trnV-GAC/rps1*), and the remaining nine repeats were spread in the CDS region. One tandem repeat was found in *rpl16* and each *ycf2* gene had four repeats (**Supplementary Table 6**).

### 3.8 Prediction of RNA-editing sites

Using Deepred-Mt, 242 high-confidence C-to-U RNA editing sites were successfully identified across 60 PCGs (**Supplementary Figure 1**; **Supplementary Table 7**). Among these, *ycf2* had the highest number of RNA editing sites (24), followed by *ycf1* and *rpoC2* (17 and 13, respectively), making them the top three genes for RNA editing. Additionally, both *ndhB* and *psaB* contained 11 spots, and *accD*, *atpA*, *matK, ndhF, psaA*, and *psbA* carried seven editing sites. In descending order, fewer editing sites were present in the PCGs, including six (*psbC* and *psbD*), five (*ndhA* and *rpoB*), four (*atpI, ccsA, ndhD, ndhG*, and *ndhK*), three (*atpE, cemA, pafII, petB*, and *psbB*), and two (*atpB, atpF, ndhC, ndhH, rbcL, rpl16, rpl2, rpl36*, and *rpoC1*). One editable site was detected in genes *atpH, clpP1, ndhI, pafI, petA, petD, petL, psaI, psdJ, psbK, psbL, psbT, psbZ, rpl20, rpl23, rpoA, rps14, rps16, rps18*, and *rps7*.

### 3.9 Comparison of IR boundaries within Thalictrum chloroplast genomes

**Figure 5:**
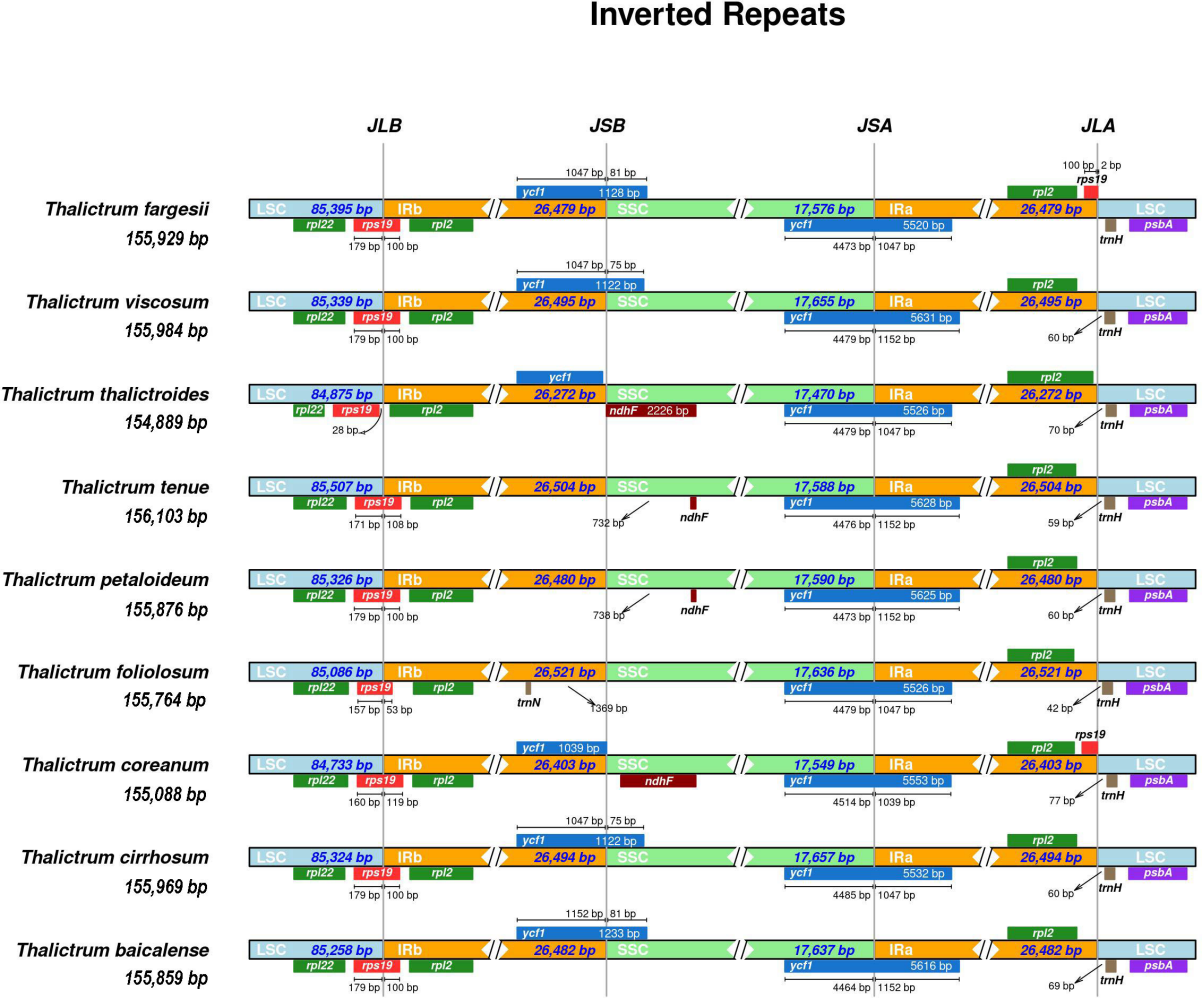
Comparison of IR boundaries of chloroplast genomes within *Thalictrum*. The junctions are the LSC-IRB, IRB-SSC, SSC-IRA, and IRA-LSC. The length inside the boxes indicates the distance between the end of the gene and the border sites. Arrows indicate the distance from the junction.

Significant contributions to variations within cp genomes among different species were observed, owing to the contraction and expansion of IR regions, which resulted in gene duplication, deletion, and pseudogene generation (Song et al., 2022). The locations of LSC/IR and SSC/IR junctions are regarded as indices of cp genome evolution (Zhang et al., 2013a). **Figure 5** shows that the size of the cp genome within the *Thalictrum* was 154,889–156,103 bp; the lengths of the LSC and SSC were 84,733–85,507 bp and 17,470–17,657 bp, respectively; and the lengths of the IR regions were 26,272–26,521 bp. The LSC/IRB border is located within *rps19* and extends 53–119 bp into the IRB region. Notably, the *rps19* gene spanned 119 bp from the LSC to IRB in the cp genome of *T. coreanum*, whereas it stretched by only 100 bp in the same direction in the *T. fargesii* plastome. In *T. thalictroides*, the LSC/IRB border is flanked by an intergenic region of 28 bp between *rps19* and the border, and the IRB/SSC border is between the partial *ycf1* and *ndhF* genes.

In the SSC region, the *ndhF* gene was larger in *T. thalictroides* than in the other species. Moreover, *T. foliolosum* lacks *ndhF*. The *ycf1* fragment gene crosses the IRB/SSC border as a pseudogene in *T. viscosum*, *T. cirrhosum*, *T. balcalense*, and *T. fargesii*. In contrast, the SSC/IRA junction was positioned on the full-length *ycf1* gene in all the species. However, the size of the overlapping *ycf1* gene at the SSC/IRA junction varies among the *Thalictrum* species. Remarkably, this gene enveloped 1,039–1,152 bp of the IRA region in all species. In addition, the IRA/LSC border was pointed between the *rpl2* and *trnH* genes and 42–77 bp from the *trnH* region toward the LSC region. Notably, exceptions were observed in *T. fargesii* and *T. coreanum*, where the *rps19* fragment was present at the edge of the IRA/LSC. The *rps19* fragment ended at the termination of the IRA region in *T. coreanum*, whereas it was 2 bp extended toward the LSC in *T. fargesii*. These results demonstrate that the IR and SC borders of the cp genomes within the *Thalictrum* are primarily similar in terms of organization, gene content, and gene order.

### 3.10 Divergence hotspot regions

**Figure 6:**
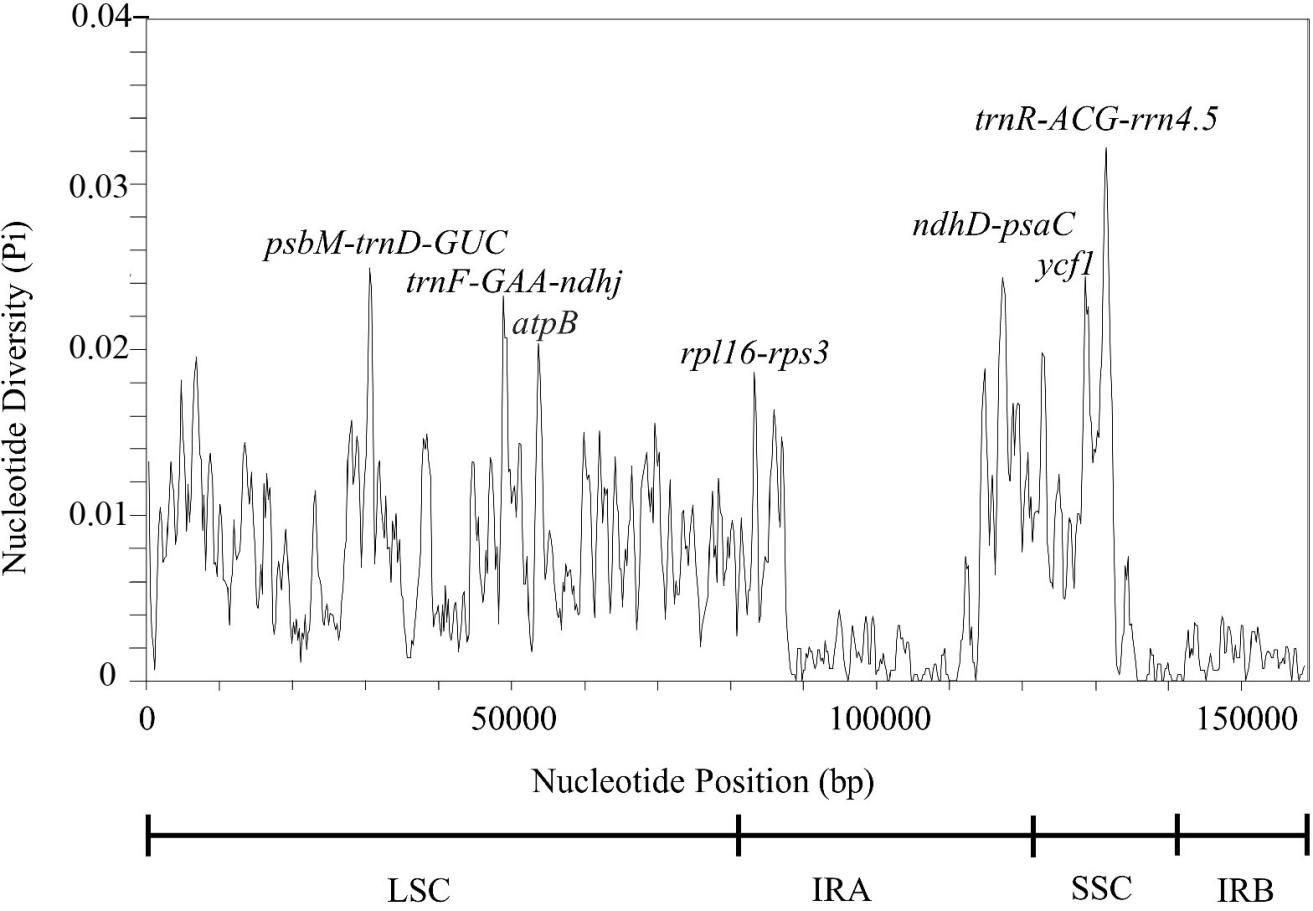
Sliding window analysis of the complete *T. fargesii* cp genome. Nucleotide diversity analysis (π value) of the complete chloroplast genome of *T. fargesii*. Window length: 600 bp; step size: 200 bp. *X*-axis: Position of the midpoint of the window. *Y*-axis: Nucleotide diversity of each window.

Highly variable chloroplast genome sequences can be used to ascertain phylogenetic relationships between species and genera (Liu et al., 2018; Wu et al., 2018). To assess the degree of sequence divergence, we computed nucleotide diversity values (*pi*) to locate hotspots; hence, the results are presented for the entire *T. fargesii* cp genome in a plot of *pi* values ranging from 0 to 0.033 (**Figure 6**). In the plot, the IR regions were shown to have a lower variability than the SSC and LSC regions. Remarkably, seven highly variable regions (*pi* > 0.02) were detected: three in the LSC (*psbM-trnD-GUC*, *trnF-GAA-ndhj*, and *atpB*), two in the SSC (*ycf1* and *trnR-ACG-rrn4.5*), and two in the IRA (*ndhD-psaC* and *rpl16-rps3*). These regions may also be employed as high-potential fragments to distinguish between different *Thalictrum* species and may help in the development of molecular markers.

### 3.11 Selection pressure on genes

In this study, we analyzed the rates of nonsynonymous (dn), synonymous (ds), and dn/ds (ω) ratios among 77 PCGs common to *Thalictrum*, and the results are shown in **Supplementary Table 8**. Among the 77 PCGs, the results of the statistical neutrality test indicated that the genes were relatively stable during the evolutionary process, and only 12 genes were found to have ω values less than 1 (**Supplementary Figure 2**). To manifest, seven genes (*atpF, matK, petG, rpl22, rps2, ycf1*, and *pafI*) were under neutral selection (ω = 1), and four genes (*atpE, ccsA, ndhG*, and *rpl20*) had ω values of precisely 0.5, indicating that the genes were under purifying selection. The remaining gene, *ndhF*, had a ω = 0.33, suggesting that it was also under purifying selection.

### 3.12 Phylogenetic analysis

**Figure 7:**
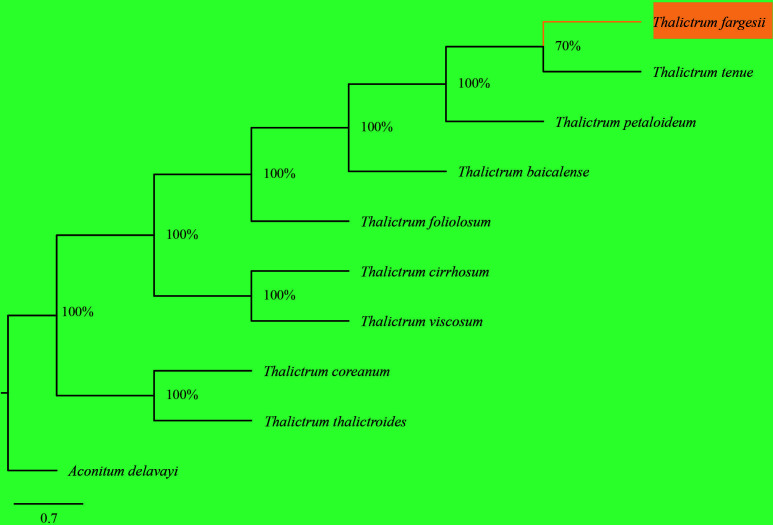
Phylogenetic relationships of *Thalictrum* inferred from maximum likelihood (ML) analysis based on whole chloroplast genomes. The numbers in each node indicate ML bootstrap values. The scale bar shows 0.7 changes.

The appearance of a complete *T. fargesii* cp genome provided us with access to sequencing data that could be used to investigate the phylogeny and, therefore, contribute to our understanding of evolutionary relationships within the *Thalictrum*. In the present study, nine species were analyzed to ascertain their phylogenetic position, and *Aconitum delavayi* (NC_038097) was chosen as an outgroup. ML analysis revealed a single phylogenetic tree with higher bootstrap values, which resulted in constructing one cluster from the complete cp genome of *Thalictrum* species divided into two clades (**Figure 7**). Hence, *Thalictrum* was strongly supported as a monophyletic group [bootstrap support (bs) = 100%]. This result indicates that *T. fargesii* is closely related to *T. tenue* and *T. petaloideum*.

### 3.13 Variations in gene content within Thalictrum

Regarding variation in gene content, we observed that *pafI* and *pafII* were found only in the cp genomes of *T. fargesii* and *T. cirrhosum* (**Supplementary Table 9**). Moreover, in the case of pseudogenes, *ycf1*, *infA*, and *rpl32* were identified in the *T. baicalense*, *T. coreanum*, *T. thalictroides*, and *T. fragesii* cp genomes, whereas the *rps19* fragment (as a pseudogene) existed only in *T. coreanum* and *T. fargesii*, indicating significant variations. The remaining species in the genus showed a relative absence of pseudogenes in their cp genomes.

### 3.14 Hotspots of variant sites in the chloroplast genome

**Figure 8:**
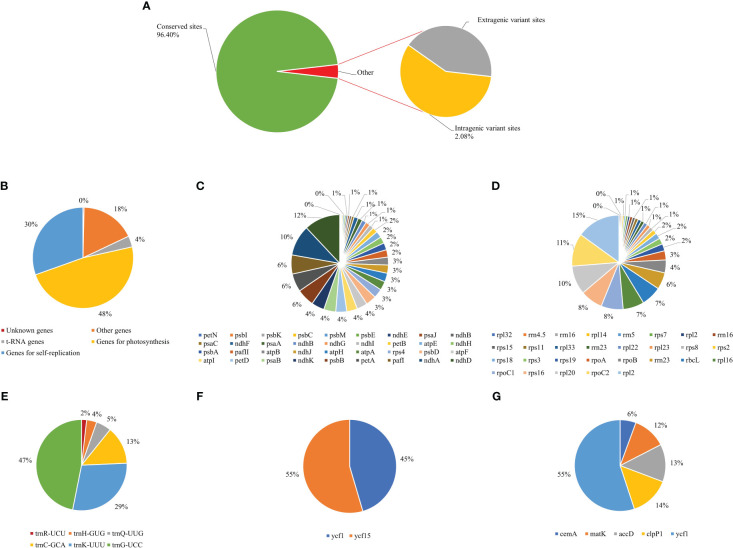
The ratio of chloroplast genome variant sites in *Thalictrum* species. **(A)** Distribution of the different variant sites. **(B)** The frequency of intragenic variant loci in different gene categories. **(C)** Frequency of variant loci among photosynthesis genes. **(D)** Frequency of variant loci among genes involved in self-replication. **(E)** Frequency of variant loci in the t-RNA genes. **(F)** Frequency of variant loci in the unknown genes. **(G)** Frequency of variant loci in the other genes.

Variant sites are the most useful for identifying genes with significant variation and can facilitate the development of molecular markers (Li and Cullis, 2023). The whole cp genomes of the nine *Thalictrum* species were compared with those of *T. fargesii*, which served as a reference to investigate variant sites. Our results showed that 3.60% of the variant sites were present, and the conserved sites accounted for 96.40%, suggesting that these sequences were highly conserved within one genus (**Supplementary Table 2**; **Figure 8**). Of the variant sites, 5,609 (3.60%) included 1.51% extragenic variant sites and 2.08% intragenic variant sites, indicating that the noncoding regions were highly conserved (**Figure 8A**). Within the intragenic sites, there were 1,500 (48%), 951 (30%), 550 (18%), 111 (4%), and 11 (0%) variant loci in the genes related to photosynthesis, self-replication, other, t-RNA, and unknown, respectively (**Figure 8B**). Furthermore, we calculated the frequency of variant sites within each category. Genes that contained more than 5% of the variant sites within each category included photosynthesis (*ndhD, ndhA, pafI, petA, psbB*, and *ndhK*; **Figure 8C**), self-replication (*rpl32, rpl33, rps18, rps19, rpl16, rps16, rpl20*, and *rpl2*; **Figure 8D**), t-RNAs (*trnQ-UUG, trnC-GCA, trnK-UUU*, and *trnG-UCC*; **Figure 8E**), unknown (*ycf1* and *yfc15*; **Figure 8F**), and others (*cemA, matK, accD, clpP1*, and *ycf1*; **Figure 8G**).

## 4 Discussion

### 4.1 The conserved genome of Thalictrum

*T. fargesii* is of considerable importance in herbal medicine in Asian countries; however, taxonomic controversies need to be resolved, which may be possible from a detailed study of its plastome genome (Li et al., 2020). Such a systemic investigation and evolutionary analysis of *T. fargesii* has not been published previously. Therefore, our study reports the first whole cp genome composition of *T. fargesii*, which provides potential evidence via phylogenomics. Previous studies on other angiosperm groups have shown that chloroplast genomes are conserved or highly polymorphic (Abdullah et al., 2020, Abdullah et al., 2021). In the present study, the cp genome was highly conserved in terms of gene order, gene content, and intron number, following the findings of many other taxa, including *Thalictrum* (Tang et al., 2015; Yu et al., 2018; Xiang et al., 2022). However, in some taxa, such as *Amorphophallus* of Araceae, some genes were lost (Liu et al., 2019), and in others, such as *Pelargonium* (Chumley et al., 2006), the structure and gene order diverged from those reported here and in most other angiosperms.

Overall, the structure and organization of the nine observed cp genomes in *Thalictrum*, including *T. fargesii*, follow the typical structure commonly found in angiosperms, except for a difference in tRNA content in *T. viscosum*, which contains 38 tRNAs instead of 37 (Park et al., 2015; Daniell et al., 2016; He et al., 2019; Morales-Briones et al., 2019; He et al., 2021; Cai et al., 2022; Zhe et al., 2023). The total length, GC content, and gene composition were almost identical in the cp genomes of all studied species, and these highly conserved features might be due to a typical evolutionary passage in *Thalictrum* (Khan et al., 2020; Villanueva-Corrales et al., 2021; Feng et al., 2022). Compared to the LSC and SSC regions, the IR region was more conserved among the cp genomes.

In addition, the ratio of nucleotide substitutions (dn/ds) and their occurrence are frequently utilized as powerful tools to elucidate the evolution of protein-encoding genes and species adaptations (Kimura, 1989; Fay and Wu, 2003). It is noteworthy that the chloroplast genome of *T. fargesii* only contains neutral selection sites and purifying sites, suggesting that these genes have conserved functions throughout their evolutionary history (Khakhlova and Bock, 2006). These results offered further evidence that the cp genomes of the *Thalictrum* are conserved.

### 4.2 Spotlights on significant molecular variations within Thalictrum

Next-generation sequencing was used to sequence the cp genomes of the ethnodrug species *T. fargesii*. The plastome of this species varies in terms of genomic structure, nucleotide diversity, structural alterations, and phylogeny with the plastomes of *Thalictrum* species (Park et al., 2015; He et al., 2019; Morales-Briones et al., 2019; He et al., 2021; Cai et al., 2022; Zhe et al., 2023). For instance, there is an apparent difference between *Thalictrum* plastomes in the IR-SC boundary areas, which causes variations in genome size (Xiang et al., 2022). IR-SC contractions or expansions into or out of neighboring single-copy regions are typically observed in angiosperm plastomes, and this process is a primary mechanism and an example of length variation in cp genomes (Kim and Lee, 2004; Yang et al., 2016; Zhang et al., 2016; Ye et al., 2018). Indeed, in our investigation, several genes, including *ycf1, rps19, ndhF, ycf15*, and *ψrpl32*, were worth examining more closely. For example, *ycf1* is duplicated in the cp genomes of several members of the *Thalictrum*, with a larger copy (*ycf1*, 5,616–5,658 bp) positioned at the SSC-IRA boundary and a fragmented copy (*ψycf1*, 1,144–1,152 bp) located at the SSC-IRB boundaries, including *T. fargesii* (Xiang et al., 2022). Other taxonomic groups also contain the pseudogene *ycf1* at the same junction (Henriquez et al., 2020; Mehmood et al., 2020). According to our findings, the shortest *ycf1* gene was present in the cp genome of *T. fargesii* (5,520 bp), with more than 5% of the variant sites within the genus. Thus, *ycf1* has enormous potential for use in developing molecular markers and has also been reported in *Fritillaria* and *Pulsatilla* (Bi et al., 2018; Li et al., 2018, Li et al., 2020).

Among such variations in cp genomes, molecular evolutionary phenomena including mutations, substitutions, selections, and random genetic drifts play a significant role. One of the molecular features known as “codon use bias” also helps unveil chloroplast and nuclear genome relationships and has been influenced by the GC content at codon locations in various organisms (Yang et al., 2018). Moreover, it is a critical feature for studying the functions of genes and contributes to its evolutionary relationship (Wang et al., 2023; Yang et al., 2023). We disclosed the presence of AT-rich intergenic regions in our present analysis, which is harmonized with the phenomenon that the overall GC content in the cp genomes of terrestrial plants is often low (Yang et al., 2017). Codon usage has a strong relationship with GC content in the codon position, and it is considered an important parameter in understanding evolutionary processes and selection pressure on genes (Zhang et al., 2013b). Our investigation revealed that of the 31 codons with RSCU values higher than one, 16 ended in U and 13 ended in A, indicating natural selection and mutation (Necşulea and Lobry, 2007). This finding suggests that *T. fargesii* prefers to employ synonymous codons with a third base, A or U, which is comparable to that of other *Thalictrum* species (Xiang et al., 2022; Zhe et al., 2023). This may be attributed to the abundance of A/T nucleotides in the angiosperm cp genomes (Abdullah et al., 2020). For instance, alanine and leucine were the most common codons in the *T. fargesii* chloroplast, which might be related to the abundance of A or T in the IR region (Chen et al., 2015), and is consistent with previous studies on the cp genomes of angiosperms (Orešič and Shalloway, 1998). *Lauraceae* exhibited a similar trend (Cao et al., 2023); however, the cp genome of *Lespedeza* showed comparable results (Somaratne et al., 2019).

Repeat sequences, particularly SSRs, are widely distributed in cp genomes, as microsatellite repeats play an essential role in gene expression, transcription control, and chromosomal construction, and display a high level of polymorphism (Qi et al., 2018; Huang et al., 2022; Xia et al., 2022). Our results support the hypothesis that cp-genome SSRs have substantial A/T bias, and this trend frequently occurs in many plants (Vieira et al., 2014; Wang et al., 2018). Similar to *T. fargesii*, mononucleotide repeats are the most abundant in *Fritillaria*, *Lilium*, and *Epimedium* (Rønsted et al., 2005; Zhang et al., 2016). Xiang et al. (2022) have reported 39 to 60 SSRs among 11 *Thalictrum* species; however, we have identified a total of 68 such repeats. In future genetic studies, we may leverage that SSRs could be used as a source of molecular markers and genomic barcodes for the *T. fargesii* cp genome. Previous studies have demonstrated that improperly combined repeating or sliding sequences can cause genomic rearrangements (Lu et al., 2017). Long repeat sequences promote cp genome rearrangement and increase the genetic diversity of a population (Guo et al., 2017; Cui et al., 2019). For example, the *ycf2* gene provides a suitable resource for genomic studies in the cp genome of *T. fargesii*, similar to those in other plant species (Cauz-Santos et al., 2017; Shen et al., 2017; Liu et al., 2018). Owing to the presence of different repeats in genes, such as *ycf1*, *ycf2*, and *rpl32*, resulting in a pseudogenized gene or loss of functions, become a significant molecular feature of cp genome in plant evolution and identification (Santos and Pereira, 2018; Park et al., 2019; Miao et al., 2022).

In higher plants, RNA editing occurs as a codon-specific event for converting cysteine (C) to uridine (U) during posttranslational modification. Therefore, this molecular process is crucial for RNA maturation to prevent unwanted mutations and to act as a signal for adaptation (Chu and Wei, 2019; Somaratne et al., 2019). Numerous plants, including *Arabidopsis*, tobacco, pea, and tomato, have RNA editing sites (Wang et al., 2016). Thus, our findings on the significant C-to-U editing mechanism in the plastid genome of *T. fargesii* can aid in detecting missense mutations in its genes (Chen et al., 2018; Small et al., 2020).

Divergent regions in cp genomes can help to identify closely related species and reveal phylogenetic information (Dong et al., 2012; Du et al., 2017). These regions have been adopted to generate barcodes for plant identification in other families of plants, including woody plants (Tan et al., 2018; Yan et al., 2023). According to our findings, *ndhD-psaC* and *rpl16-rps3* were found to be divergent hotspots in the cp genome of *T. fargesii*, and have also been reported in other studies on the plastome of *Thalictrum* (Xiang et al., 2022). The same phenomenon has been witnessed for numerous other taxa such as *Lagerstroemia* and *Adrinandra* (Cui et al., 2019; Dong et al., 2021; Nguyen et al., 2021; Ren et al., 2022; Yang et al., 2022). In addition, these mutational hotspots might aid researchers in studying population genetics and identifying *Thalictrum* species.

To evaluate the selection pressure on genes and genomes, dn and ds values are important markers in the cp genome. Selection pressure plays a vital role in the adaptation to different environments (Yang and Nielsen, 2000; Gao et al., 2019). Several genes in *T. fargesii* were found to have a lower dn/ds ratio, suggesting that they are likely to undergo disadvantageous non-synonymous substitutions and purifying selections (Sheikh-Assadi et al., 2022). In *T. coreanum*, *T. foeniculaceum*, *T. foliolosum*, and *T. thalictroides*, the *ndhG* gene was significant under positive selection, with a lower codon bias index, suggesting a lower expression level *in vivo* (Xiang et al., 2022). Moreover, they reported that *atpF*, *petG*, *rpl20*, and *rpl22* were under positive selection. In contrast, in the present study, the *ndhG* and *rpl20* were observed under purifying selection, and *atpF*, *petG*, and *rpl22* were supported by neutral selection in *T. fargessii*. Thus, purifying selection might be a result of an evolutionary process that preserves the adaptive behavior of this species.

Interestingly, the *pafI* and *rps19* genes in the plastome of *T. fargesii* exhibited more than 5% variation within the *Thalictrum*, which was consistent with the findings of Li et al. (2014). In addition, the *pafI* gene contains two mono-type (10 bp each) SSR repeats and a 39-bp forward repeat at one of its introns, similar to the findings elucidated by Kurt et al. (2023). With the meaningful characteristic architecture of the IRA-LSC, the distance between the *rpl2* and t*rnH* genes was markedly different from that of other species in the genus. Moreover, similar to the phenomenon observed in *Fritillaria*, the presence of the *rps19* fragment at the IRA-LSC junction in the *T. fargesii* cp genome and its distance from *trnH* make this region a probable string for marker design (Li et al., 2014). Cumulatively, these findings provide molecular evidence for differentiating the ethnodrug plant *T. fargesii* from other species of *Thalictrum*.

### 4.3 Insights into the phylogeny of Thalictrum

The phylogeny and classification of *Thalictrum* have long been debated, and distinguishing *T. fargesii* plants from plants with similar morphological traits remains unclear (Xiang et al., 2022). Plastid genome sequences have proven useful for phylogenetic investigations of angiosperms (Jansen et al., 2007; Huang et al., 2014; Kim et al., 2015; Li et al., 2019). This approach is valuable for classifying organisms at lower taxonomic levels (He et al., 2012; Zhang et al., 2016). Previous phylogenetic studies of the genus *Thalictrum* have used traditional approaches to classify this genus that includes *rpl16* intron and *ndhC-trnV-UAC* by Soza et al., (2012, 2013), and *ndhA* intron, *trnLUAA-trnF-GAA*, *rpl32-trnL-UAG*, and *rbcL* by Wang et al. (2019). Moreover, Xiang et al. (2022) found that 116 IGS regions had stronger support than 114 gene sequences and revealed that the closest phylogenetic relationship existed within *T. minus* and *T. aquilegiifolium*, and *T. coreanum* and *T. thalictroides*. Several studies have uncovered that close relationships were present between *T. minus* and *T. tenue* (He et al., 2019; Lin et al., 2021; Pu et al., 2022), *T. tenue* and *T. petaloideum* (He et al., 2019; Pu et al., 2022), *T. minus* and *T. petaloideum* (He et al., 2019; Pu et al., 2022), *T. minus* and *T. aquilegiifolium* (Cai et al., 2022; Michimoto et al., 2022; Xiang et al., 2022), *T. viscosum* and *T. cirrhosum* (Cai et al., 2022; Michimoto et al., 2022), and *T. coreanum* and *T. thalictroides* (He et al., 2021; Lin et al., 2021; Michimoto et al., 2022; Xiang et al., 2022) based on all sequences and whole cp genome. However, none of their circumscribed relationships have uncovered the *T. fargesii* status within this genus. The availability of complete cp genomes of *T. fargesii*, as investigated by the present authors, provides sufficient information to establish phylogenetic relationships among the same taxa. According to their results, *T. fargesii* is closely related to *T. tenue* and *T. petaloideum*, supporting the conclusion of an earlier study that *T. baicalense* is closely related to *T. tenue, T. minus*, and *T. petaloideum* (He et al., 2021). Interestingly, *T. viscosum* has been reported to be closely related to *T. cirrhosum* and *T. foeniculaceum* (Cai et al., 2022). Conferring with more *Thalictrum* taxon samples, our phylogenetic studies of cp genome sequences, reported for the first time, revealed significantly enriched phylogenetic resolution and provided robust inferences of intraspecific relationships. This finding expands the scope of future research on the correlation between these mutations in the cp genome in terms of genetic evolution and speciation in this species. Further studies should entail selecting more samples from wild populations based on their ecotypes and collecting more extensive data from both the nuclear and cp genomes to establish more detailed evidence of phylogenetics and species evolution.

## 5 Conclusion

Altogether, our findings revealed the complete cp genome of *T. fargesii* using the Illumina HiSeq platform and a comparative analysis with those of other *Thalictrum* species provides a fundamental reference for phylogenetic studies. The chloroplast genomes of *Thalictrum* were compared, and although they were found to be highly conserved in terms of structure, organization, gene order, IR boundaries, and gene content, we have reported distinguishable features of *T. fargesii*. In particular, two features have clearly demarcated the cp genome of *T. fargesii* within the genus, which is the length of pseudogenes and their distance from IR/SC borders. Moreover, *pafI* and *rps19* had highly variable sites in the cp genome of *T. fargesii* compared with other species in the genus. In addition, the divergent regions identified in the present study *via* nucleotide divergence analysis could help design molecular markers to identify this ethnodrug and its contaminants. Phylogenetic analysis revealed close relationships among *T. fargesii*, *T. tenue*, and *T. petaloidium*. The aforementioned analytical findings significantly contribute to the understanding of the cp genome of *T. fargesii* and provide genomic information to aid taxonomic identification and phylogenetic relationships within the *Thalictrum* species. In conclusion, our study provides powerful resources and valuable scientific references for identifying *T. fargesii* plants for the safety and effectiveness of ethnodrug use and contributes to the bioprospecting and conservation of this species.

## Data availability statement

The datasets presented in this study can be found in online repositories. The names of the repository/repositories and accession number(s) can be found in the article/**Supplementary Material**.

## Author contributions

SC: Conceptualization, Data curation, Formal analysis, Investigation, Methodology, Resources, Software, Supervision, Validation, Visualization, Writing – original draft, Writing – review & editing, Funding acquisition, Project administration. FS: Conceptualization, Data curation, Formal analysis, Investigation, Methodology, Resources, Software, Supervision, Validation, Visualization, Writing – original draft, Writing – review & editing. LAk: Data curation, Investigation, Methodology, Writing – original draft, Writing – review & editing. LAo: Data curation, Formal analysis, Funding acquisition, Investigation, Methodology, Project administration, Writing – review & editing. YZ: Conceptualization, Funding acquisition, Investigation, Project administration, Resources, Supervision, Writing – review & editing. YQ: Methodology, Validation, Resources, Visualization, Writing – review & editing.

## Supplementary material

Supplementary Figure 1 Distribution of RNA editing sites among the genes in the cp genome of *T. fargesii*.

Supplementary Figure 2 Genes under selective pressure in *Thalictrum*.
