# Supplementary figures and images for: The first complete chloroplast genome of Thalictrum fargesii: insights into phylogeny and species identification
Source: Front Plant Sci. 2024 Apr 29;15:1356912. doi: 10.3389/fpls.2024.1356912 (PMC11092384; doi:10.3389/fpls.2024.1356912)

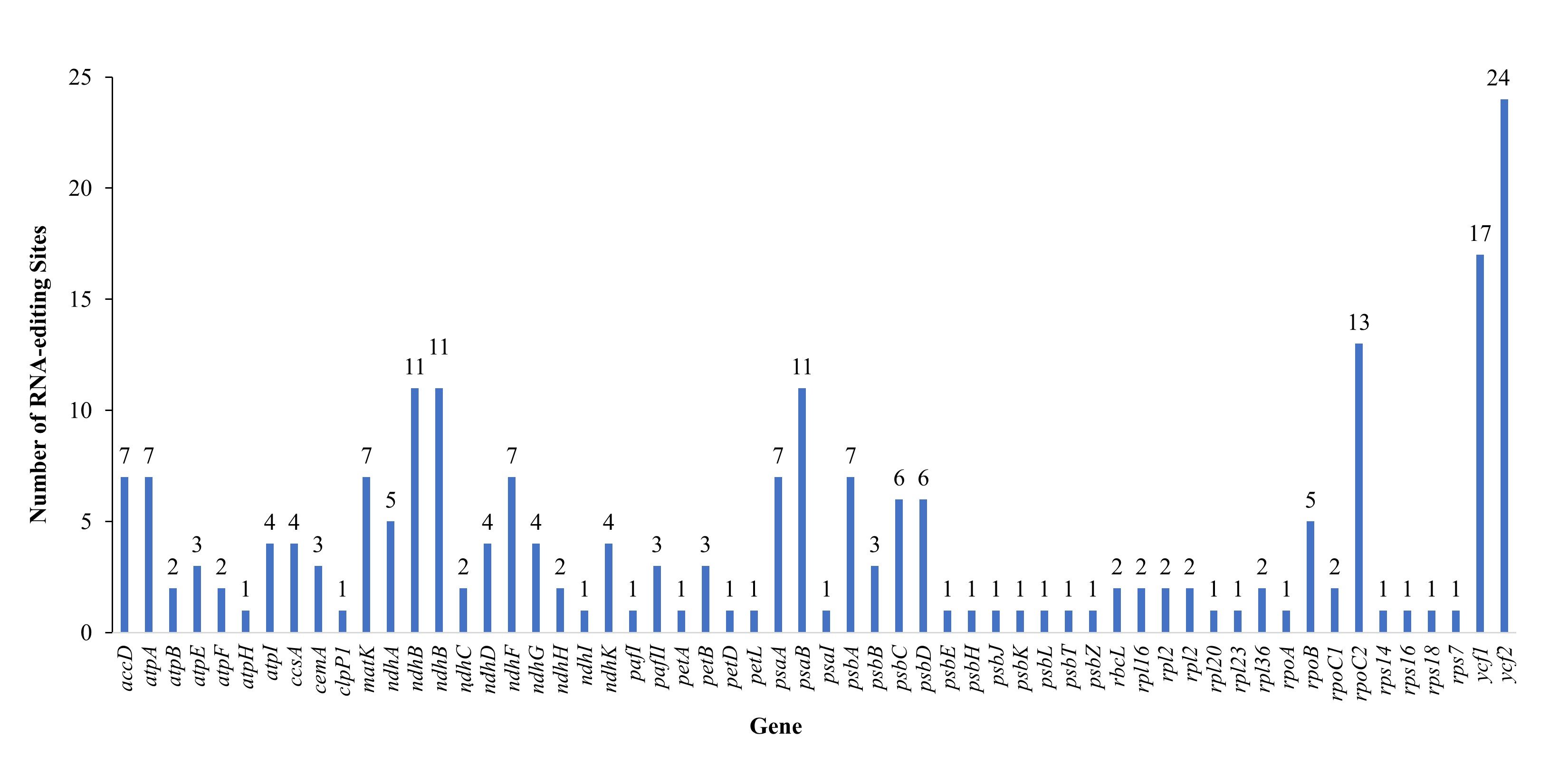

Supplement: Supplementary Figure 1 — Distribution of RNA editing sites among the genes in the cp genome of T. fargesii. [file Image_1.jpeg]

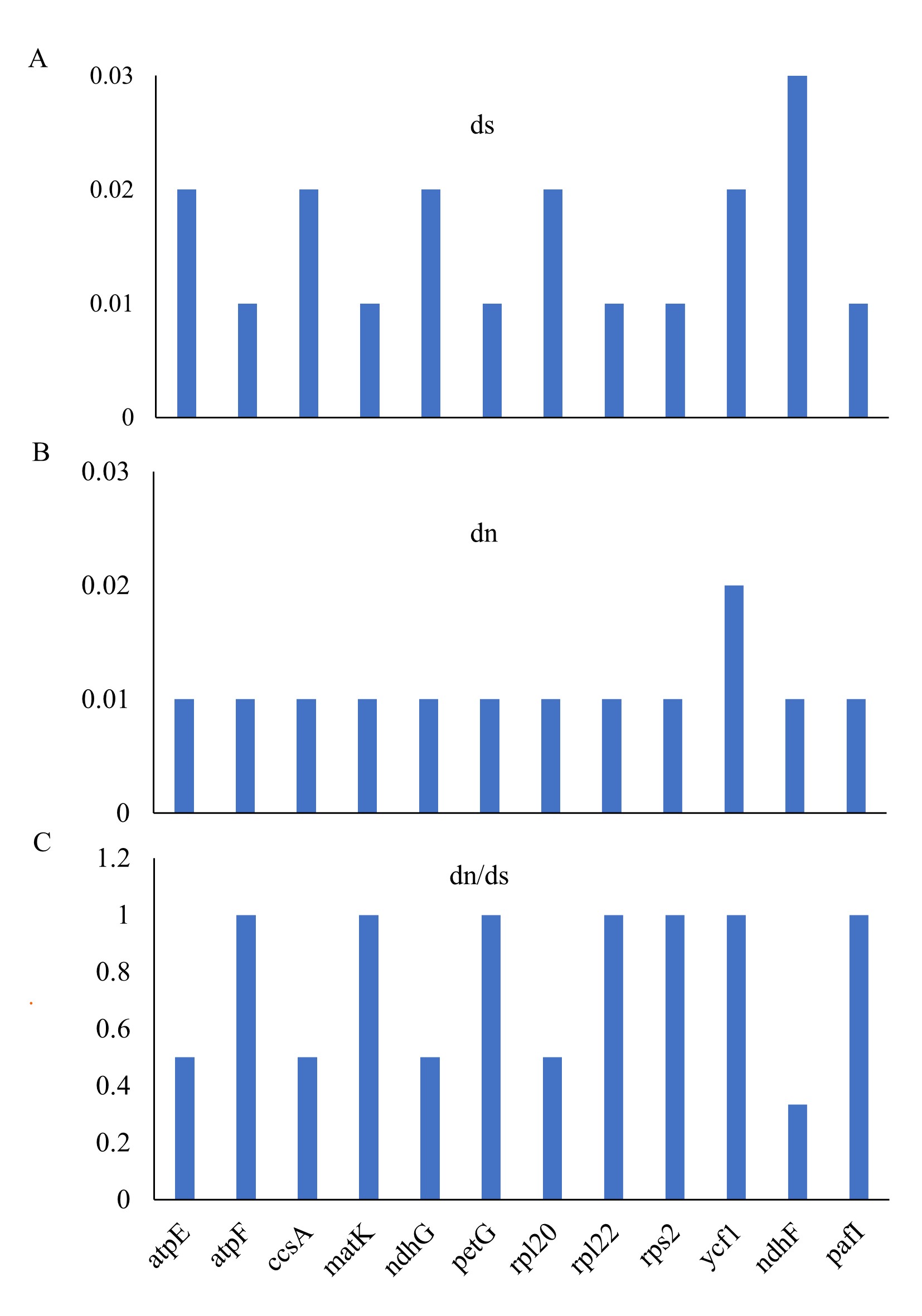

Supplement: Supplementary Figure 2 — Genes under selective pressure in Thalictrum. [file Image_2.jpeg]
