# Supplementary material for: The first complete chloroplast genome of Thalictrum fargesii: insights into phylogeny and species identification
Source: Front Plant Sci. 2024 Apr 29;15:1356912. doi: 10.3389/fpls.2024.1356912 (PMC11092384; doi:10.3389/fpls.2024.1356912)
Supplement: Supplementary file 3 [file Table_1.docx]

| **Species name** | **Genome size**  **(bp)** | **LSC**  **size**  **(bp)** | **SSC**  **Size**  **(bp)** | | **IR**  **Size (bp, each)** | | **Genes** | **PCGs** | **tRNAs** | **rRNAs** | **GC%** | **GenBank accession number** | **References** |
| --- | --- | --- | --- | --- | --- | --- | --- | --- | --- | --- | --- | --- | --- |
| *Thalictrum baicalense* | 155,859 | 85,258 | | 17,637 | | 26,482 | 131 | 86 | 37 | 8 | 38 | MW133265 | He et al., 2021 |
| *Thalictrum cirrhosum* | 155,969 | 85,324 | | 17,657 | | 26,494 | 134 | 89 | 37 | 8 | 38 | NC_061927 | Zhe et al., 2023 |
| *Thalictrum coreanum* | 155,088 | 84,733 | | 17,549 | | 26,403 | 133 | 88 | 37 | 8 | 38 | KM206568 | Park et al., 2015 |
| *Thalictrum foliolosum* | 155,764 | 85,086 | | 17,636 | | 26,521 | 128 | 83 | 37 | 8 | 38 | NC_058920 | unpublished |
| *Thalictrum petaloideum* | 155,876 | 85,326 | | 17,590 | | 26,480 | 130 | 85 | 37 | 8 | 38 | MK253449 | He et al., 2019 |
| *Thalictrum tenue* | 156,103 | 85,507 | | 17,588 | | 26,504 | 130 | 85 | 37 | 8 | 38 | MK253448 | He et al., 2019 |
| *Thalictrum thalictroides* | 154,889 | 84,875 | | 17,470 | | 26,272 | 134 | 89 | 37 | 8 | 38 | MH092834 | Morales‐Briones et al., 2019 |
| *Thalictrum viscosum* | 155,984 | 85,339 | | 17,655 | | 26,495 | 134 | 88 | 38 | 8 | 38 | NC_058831 | Cai et al., 2022 |
| *Thalictrum fargesii* | 155,929 | 85,395 | | 17,576 | | 26,479 | 133 | 88 | 37 | 8 | 38 | ON868919.1 | Presenting authors |

**Supplementary Table 1.** Basic genomic organizations of nine species of *Thalictrum* used in this study including *T. fargesii*
