# Supplementary material for: The first complete chloroplast genome of Thalictrum fargesii: insights into phylogeny and species identification
Source: Front Plant Sci. 2024 Apr 29;15:1356912. doi: 10.3389/fpls.2024.1356912 (PMC11092384; doi:10.3389/fpls.2024.1356912)
Supplement: Supplementary file 4 [file Table_2.docx]

**Supplementary Table 2.** Distribution of variant sites within *Thalictrum* species where *T. fargesii* as reference

| **Gene** | **Start position** | **End position** | **Size (bp)** | **Variant loci intervals** | **Number of variant loci** | **Ratio of intra-genic variant loci** |
| --- | --- | --- | --- | --- | --- | --- |
| *trnH-GUG* | 60 | 134 | 74 | 68-82 | 4 | 5.405% |
| *psbA* | 248 | 1309 | 1061 | 249-551 | 35 | 3.299% |
| *trnK-UUU* | 1566 | 4169 | 2603 | 1558-1858 | 32 | 1.229% |
| *matK* | 1865 | 3394 | 1529 | 1866-3377 | 65 | 4.251% |
| *rps16* | 4882 | 5975 | 1093 | 4883-5965 | 73 | 6.679% |
| *trnQ-UUG* | 7015 | 7086 | 71 | 7045-7077 | 6 | 8.451% |
| *psbK* | 7421 | 7606 | 185 | 7445-7598 | 5 | 2.703% |
| *psbI* | 8021 | 8131 | 110 | 8029-8058 | 4 | 3.636% |
| *trnS-GCU* | 8267 | 8354 | 87 | 0 | 0 | 0.000% |
| *trnG-UCC* | 8753 | 9526 | 773 | 8755-9450 | 52 | 6.727% |
| *trnR-UCU* | 9713 | 9784 | 71 | 9742-9743 | 2 | 2.817% |
| *atpA* | 9910 | 11433 | 1523 | 9924-11432 | 44 | 2.889% |
| *atpF* | 11500 | 12775 | 1275 | 11517-12758 | 53 | 4.157% |
| *atpH* | 13233 | 13478 | 245 | 13262-13470 | 43 | 17.551% |
| *atpI* | 14461 | 15204 | 743 | 14523-14942 | 53 | 7.133% |
| *rps2* | 15412 | 16122 | 710 | 15435-16090 | 15 | 2.113% |
| *rpoC2* | 16339 | 20472 | 4133 | 16347-20316 | 107 | 2.589% |
| *rpoC1* | 20645 | 23443 | 2798 | 20685-23398 | 72 | 2.573% |
| *rpoB* | 23470 | 26682 | 3212 | 23483-26671 | 42 | 1.308% |
| *trnC-GCA* | 27922 | 27992 | 70 | 27925-27992 | 15 | 21.429% |
| *petN* | 28818 | 28907 | 89 | 28839-28889 | 2 | 2.247% |
| *psbM* | 30121 | 30225 | 104 | 30133-30109 | 7 | 6.731% |
| *trnD-GUC* | 31375 | 31448 | 73 | 4 | 0 | 0.000% |
| *trnY-GUA* | 31858 | 31941 | 83 | 0 | 0 | 0.000% |
| *trnE-UUC* | 32001 | 32073 | 72 | 0 | 0 | 0.000% |
| *trnT-GGU* | 32803 | 32874 | 71 | 0 | 0 | 0.000% |
| *psbD* | 34171 | 35232 | 1061 | 34175-35075 | 52 | 4.901% |
| *psbC* | 35180 | 36601 | 1421 | 35245-35863 | 6 | 0.422% |
| *psbC* | 35216 | 36601 | 1385 | 0 | 0 | 0.000% |
| *trnS-UGA* | 36800 | 36892 | 92 | 0 | 0 | 0.000% |
| *psbZ* | 37248 | 37436 | 188 | 2 | 0 | 0.000% |
| *trnG-GCC_* | 37784 | 37854 | 70 | 0 | 0 | 0.000% |
| *trnfM-CAU* | 38033 | 38106 | 73 | 0 | 0 | 0.000% |
| *rps14* | 38268 | 38570 | 302 | 0 | 0 | 0.000% |
| *psaB* | 38696 | 40900 | 2204 | 38697-39852 | 60 | 2.722% |
| *psaA* | 40926 | 43178 | 2252 | 41154-43108 | 22 | 0.977% |
| *pafI* | 43796 | 45772 | 1976 | 44128-45765 | 95 | 4.808% |
| *trnS-GGA* | 46323 | 46409 | 86 | 0 | 0 | 0.000% |
| *rps4* | 46712 | 47317 | 605 | 46182-47315 | 46 | 7.603% |
| *trnT-UGU* | 47702 | 47774 | 72 | 0 | 0 | 0.000% |
| *trnL-UAA* | 49045 | 49094 | 49 | 0 | 0 | 0.000% |
| *trnF-GAA* | 49493 | 49565 | 72 | 0 | 0 | 0.000% |
| *ndhJ* | 50094 | 50570 | 476 | 50120-50499 | 41 | 8.613% |
| *ndhK* | 50710 | 51393 | 683 | 50714-51359 | 64 | 9.370% |
| *ndhC* | 51443 | 51805 | 362 | 2 | 0 | 0.000% |
| *trnV-UAC* | 52743 | 53410 | 667 | 0 | 0 | 0.000% |
| *trnM-CAU* | 53599 | 53671 | 72 | 0 | 0 | 0.000% |
| *atpE* | 53892 | 54293 | 401 | 53896-54277 | 32 | 7.980% |
| *atpB* | 54290 | 55786 | 1496 | 54368-55739 | 39 | 2.607% |
| *rbcL* | 56521 | 57948 | 1427 | 56662-57888 | 66 | 4.625% |
| *accD* | 58694 | 60163 | 1469 | 58751-60153 | 73 | 4.969% |
| *psaI* | 60839 | 60949 | 110 | 2 | 0 | 0.000% |
| *pafII* | 61381 | 61935 | 554 | 61403-61926 | 37 | 6.679% |
| *cemA* | 62733 | 63422 | 689 | 62737-63386 | 31 | 4.499% |
| *petA* | 63582 | 64550 | 968 | 63647-64517 | 90 | 9.298% |
| *psbJ* | 64987 | 65109 | 122 | 0 | 0 | 0.000% |
| *psbL* | 65229 | 65345 | 116 | 0 | 0 | 0.000% |
| *psbF* | 65368 | 65487 | 119 | 0 | 0 | 0.000% |
| *psbE* | 65497 | 65748 | 251 | 65525-65738 | 8 | 3.187% |
| *petL* | 66827 | 66922 | 95 | 0 | 0 | 0.000% |
| *petG* | 67103 | 67216 | 113 | 0 | 0 | 0.000% |
| *trnW-CCA* | 67361 | 67434 | 73 | 0 | 0 | 0.000% |
| *trnP-UGG* | 67627 | 67700 | 73 | 0 | 0 | 0.000% |
| *psaJ* | 68138 | 68272 | 134 | 68187-68272 | 9 | 6.716% |
| *rpl33* | 68703 | 68909 | 206 | 68707-68897 | 11 | 5.340% |
| *rps18* | 69094 | 69399 | 305 | 69129-69397 | 19 | 6.230% |
| *rpl20* | 69686 | 70039 | 353 | 69689-70027 | 94 | 26.629% |
| *clpP1* | 71106 | 73189 | 2083 | 71144-73142 | 78 | 3.745% |
| *psbB* | 73599 | 75125 | 1526 | 73615-75075 | 88 | 5.767% |
| *psbT* | 75308 | 75409 | 101 | 0 | 0 | 0.000% |
| *pbf1* | 75502 | 75633 | 131 | 0 | 0 | 0.000% |
| *psbH* | 75736 | 75957 | 221 | 0 | 0 | 0.000% |
| *petB* | 76078 | 77531 | 1453 | 76210-77527 | 26 | 1.789% |
| *petD* | 77729 | 78934 | 1205 | 77746-78946 | 55 | 4.564% |
| *rpoA* | 79117 | 80136 | 1019 | 79125-80121 | 30 | 2.944% |
| *rps11* | 80202 | 80618 | 416 | 80292-80532 | 10 | 2.404% |
| *rpl36* | 80731 | 80844 | 113 | 0 | 0 | 0.000% |
| *infA* | 81028 | 81156 | 128 | 0 | 0 | 0.000% |
| *rps8* | 81331 | 81729 | 398 | 81367-81683 | 13 | 3.266% |
| *rpl14* | 81935 | 82303 | 368 | 81941-82265 | 5 | 1.359% |
| *rpl16* | 82435 | 83800 | 1365 | 82606-83544 | 69 | 5.055% |
| *rps3* | 83962 | 84618 | 656 | 84010-84614 | 20 | 3.049% |
| *rpl22* | 84603 | 85148 | 545 | 84622-85133 | 12 | 2.202% |
| *rps19* | 85209 | 85487 | 278 | 85213-85431 | 23 | 8.273% |
| *rpl2* | 85553 | 87035 | 1482 | 85558-87023 | 143 | 9.649% |
| *rpl23* | 87054 | 87335 | 281 | 87055-87280 | 12 | 4.270% |
| *trnI-CAU* | 87501 | 87574 | 73 | 0 | 0 | 0.000% |
| *ycf2* | 87643 | 94557 | 6914 | 87650-94424 | 31 | 0.448% |
| *ycf15* | 94622 | 95211 | 589 | 94630-95121 | 6 | 1.019% |
| *trnL-CAA* | 95574 | 95654 | 80 | 0 | 0 | 0.000% |
| *ndhB* | 96222 | 98457 | 2235 | 96570-98331 | 22 | 0.984% |
| *rps7* | 98782 | 99249 | 467 | 0 | 0 | 0.000% |
| *rps12* | 99303 | 70958 | -28345 | 0 | 0 | 0.000% |
| *trnV-GAC* | 101981 | 102052 | 71 | 0 | 0 | 0.000% |
| *rrn16* | 102280 | 103770 | 1490 | 102656-103745 | 9 | 0.604% |
| *trnI-GAU* | 104065 | 105065 | 1000 | 0 | 0 | 0.000% |
| *trna-UGC* | 105130 | 106002 | 872 | 0 | 0 | 0.000% |
| *rrn23* | 106155 | 108963 | 2808 | 106583-108152 | 11 | 0.392% |
| *rrn4.5* | 109062 | 109164 | 102 | 0 | 0 | 0.000% |
| *rrn5* | 109388 | 109508 | 120 | 0 | 0 | 0.000% |
| *trnR-ACG* | 109756 | 109829 | 73 | 0 | 0 | 0.000% |
| *trnN-GUU* | 110426 | 110497 | 71 | 0 | 0 | 0.000% |
| *ycf1* | 110820 | 111947 | 1127 | 111408-111840 | 5 | 0.444% |
| *ndhF* | 112599 | 114083 | 1484 | 112733-114039 | 21 | 1.415% |
| *rpl32* | 114747 | 114825 | 78 | 11475-114808 | 4 | 5.128% |
| *trnL-UAG* | 115245 | 115324 | 79 | 0 | 0 | 0.000% |
| *ccsA* | 115437 | 116414 | 977 | 115462-116402 | 34 | 3.480% |
| *ndhD* | 116639 | 118150 | 1511 | 116739-118133 | 180 | 11.913% |
| *psaC* | 118277 | 118522 | 245 | 118279-118519 | 12 | 4.898% |
| *ndhE* | 118772 | 119077 | 305 | 118778-119062 | 8 | 2.623% |
| *ndhG* | 119308 | 119844 | 536 | 119308-119844 | 22 | 4.104% |
| *ndhI* | 120223 | 120762 | 539 | 120236-120755 | 22 | 4.082% |
| *ndhA* | 120841 | 122974 | 2133 | 120851-122966 | 153 | 7.173% |
| *ndhH* | 122976 | 124157 | 1181 | 122985-124128 | 33 | 2.794% |
| *rps15* | 124302 | 124559 | 257 | 124325-124559 | 9 | 3.502% |
| *ycf1* | 124970 | 130489 | 5519 | 124970-130486 | 303 | 5.490% |
| *trnN-GUU* | 130812 | 130883 | 71 | 0 | 0 | 0.000% |
| *trnR-ACG* | 131480 | 131553 | 73 | 0 | 0 | 0.000% |
| *rrn5* | 131801 | 131921 | 120 | 131818-131891 | 5 | 4.167% |
| *rrn4.5* | 132145 | 132247 | 102 | 132190-132198 | 4 | 3.922% |
| *rrn23* | 132346 | 135154 | 2808 | 132357-135083 | 55 | 1.959% |
| *trna-UGC* | 135307 | 136179 | 872 | 0 | 0 | 0.000% |
| *trnI-GAU* | 136244 | 137244 | 1000 | 0 | 0 | 0.000% |
| *rrn16* | 137539 | 139029 | 1490 | 137609-138710 | 4 | 0.268% |
| *trnV-GAC* | 139257 | 139328 | 71 | 0 | 0 | 0.000% |
| *rps7* | 142060 | 142527 | 467 | 142357-142475 | 7 | 1.499% |
| *ndhB* | 142852 | 145087 | 2235 | 142933-144659 | 11 | 0.492% |
| *trnL-CAA* | 145655 | 145735 | 80 | 0 | 0 | 0.000% |
| *ycf15* | 146098 | 146687 | 589 | 0 | 0 | 0.000% |
| *ycf2* | 146752 | 153651 | 6899 | 147023-153499 | 62 | 0.899% |
| *trnI-CAU* | 153735 | 153808 | 73 | 0 | 0 | 0.000% |
| *rpl23* | 153974 | 154255 | 281 | 0 | 0 | 0.000% |
| *rpl2* | 154274 | 155756 | 1482 | 154337-155536 | 7 | 0.472% |
