# Supplementary material for: The first complete chloroplast genome of Thalictrum fargesii: insights into phylogeny and species identification
Source: Front Plant Sci. 2024 Apr 29;15:1356912. doi: 10.3389/fpls.2024.1356912 (PMC11092384; doi:10.3389/fpls.2024.1356912)
Supplement: Supplementary file 5 [file Table_3.docx]

**Supplementary Table 3.** Distribution of introns among the genes in cp genome of *T. fargesii*

| **SL** | **Gene name** | **Genome location** | **Size (bp)** | **Region** |
| --- | --- | --- | --- | --- |
| 1 | *trnK-UUU* | 1601..4132 | 2532 | LSC |
| 2 | *rps16- intron I* | 5078..5931 | 854 |  |
|  | *rps16-intron II* | 5082..5935 | 703 |  |
| 4 | *trnG-UCC* | 8776..9478 | 721 |  |
| 5 | *atpF* | 11910..12630 | 595 |  |
| 6 | *trnV-UAC-intron I* | 52778..53372 | 593 |  |
|  | *trnV-UAC-intron II* | 52780..53372 | 806 |  |
| 8 | *petB* | 76084..76889 | 723 |  |
| 9 | *petD* | 77737..78459 | 958 |  |
| 10 | *rpl16* | 82834..83791 | 958 |  |
| 11 | *rpl2* | 85987..86644 | 658 | IRA |
| 12 | *ndhB* | 96978..97680 | 703 |  |
| 13 | *rps12* | 99329..99863 | 535 |  |
| 14 | *trnI-GAU* | 104102..105030 | 929 |  |
| 15 | *trnA-UGC* | 105168..105967 | 800 |  |
| 16 | *rrn23* | 108765..108963 | 199 |  |
| 17 | *trnA-UGC* | 135342..136141 | 800 | IRB |
| 18 | *trnI-GAU* | 136279..137207 | 929 |  |
| 19 | *rps12* | 141446..141980 | 535 |  |
| 20 | *ndhB* | 143629..144331 | 703 |  |
| 21 | *rpl2* | 154665..155322 | 658 |  |
