# Supplementary material for: The first complete chloroplast genome of Thalictrum fargesii: insights into phylogeny and species identification
Source: Front Plant Sci. 2024 Apr 29;15:1356912. doi: 10.3389/fpls.2024.1356912 (PMC11092384; doi:10.3389/fpls.2024.1356912)
Supplement: Supplementary file 8 [file Table_6.docx]

**Supplementary** **Table 6.** Distribution and composition, location of tandem repeats in cp genome of *T. fargesii*

| **No.** | **Position**  **(Start-End)** | **Period Size** | **Copy Number** | **Consensus Size** | **Percent Matches** | **Percent Indels** | **Score** | **A** | **C** | **G** | **T** | **Entropy (0-2)** | **Location*** |
| --- | --- | --- | --- | --- | --- | --- | --- | --- | --- | --- | --- | --- | --- |
| 1 | [26751-26791](https://tandem.bu.edu/trf/output/tmpjfkfuxg0.2.7.7.80.10.50.500.1.txt.html#26751--26791,21,2.0,21,1) | 21 | 2.0 | 21 | 90 | 0 | 64 | 29 | 12 | 29 | 29 | 1.93 | *rpoB/trnC-GCA (IGS)* |
| 2 | [29747-29786](https://tandem.bu.edu/trf/output/tmpjfkfuxg0.2.7.7.80.10.50.500.1.txt.html#29747--29786,18,2.2,18,2) | 18 | 2.2 | 18 | 100 | 0 | 80 | 30 | 0 | 5 | 65 | 1.14 | *petN/psbM* (IGS) |
| 3 | [29772-29824](https://tandem.bu.edu/trf/output/tmpjfkfuxg0.2.7.7.80.10.50.500.1.txt.html#29772--29824,23,2.5,20,3) | 23 | 2.5 | 20 | 77 | 17 | 52 | 37 | 5 | 1 | 54 | 1.35 | *petN/psbM* (IGS) |
| 4 | [32982-33041](https://tandem.bu.edu/trf/output/tmpjfkfuxg0.2.7.7.80.10.50.500.1.txt.html#32982--33041,19,2.9,21,4) | 19 | 2.9 | 21 | 85 | 12 | 88 | 41 | 0 | 0 | 58 | 0.98 | *trnT-GGU/psbD* (IGS) |
| 5 | [37510-37534](https://tandem.bu.edu/trf/output/tmpjfkfuxg0.2.7.7.80.10.50.500.1.txt.html#37510--37534,12,2.1,12,6) | 12 | 2.1 | 12 | 100 | 0 | 50 | 44 | 16 | 0 | 40 | 1.47 | *psbZ/trnG-GCC* (IGS) |
| 6 | [37677-37740](https://tandem.bu.edu/trf/output/tmpjfkfuxg0.2.7.7.80.10.50.500.1.txt.html#37677--37740,2,35.5,2,7) | 2 | 35.5 | 2 | 72 | 20 | 52 | 46 | 1 | 0 | 51 | 1.10 | *psbZ/trnG-GCC* (IGS) |
| 7 | [56386-56410](https://tandem.bu.edu/trf/output/tmpjfkfuxg0.2.7.7.80.10.50.500.1.txt.html#56386--56410,13,1.9,13,8) | 13 | 1.9 | 13 | 100 | 0 | 50 | 40 | 0 | 20 | 40 | 1.52 | *atpB/rbcL* (IGS) |
| 8 | [67437-67463](https://tandem.bu.edu/trf/output/tmpjfkfuxg0.2.7.7.80.10.50.500.1.txt.html#67437--67463,13,2.1,13,9) | 13 | 2.1 | 13 | 100 | 0 | 54 | 37 | 22 | 0 | 40 | 1.54 | *trnW-CCA/ trnP-UGG* (IGS) |
| 9 | [67873-67920](https://tandem.bu.edu/trf/output/tmpjfkfuxg0.2.7.7.80.10.50.500.1.txt.html#67873--67920,22,2.2,22,11) | 22 | 2.2 | 22 | 92 | 7 | 80 | 56 | 0 | 0 | 43 | 0.99 | *trnP-UGG/ psaJ* (IGS) |
| 10 | [67849-67903](https://tandem.bu.edu/trf/output/tmpjfkfuxg0.2.7.7.80.10.50.500.1.txt.html#67849--67903,27,2.0,28,12) | 27 | 2.0 | 28 | 85 | 3 | 76 | 60 | 3 | 0 | 36 | 1.15 | *trnP-UGG/ psaJ* (IGS) |
| 11 | [83006-83043](https://tandem.bu.edu/trf/output/tmpjfkfuxg0.2.7.7.80.10.50.500.1.txt.html#83006--83043,19,2.0,19,13) | 19 | 2.0 | 19 | 100 | 0 | 76 | 47 | 0 | 5 | 47 | 1.24 | *rpl16* (CDS) |
| 12 | [90228-90296](https://tandem.bu.edu/trf/output/tmpjfkfuxg0.2.7.7.80.10.50.500.1.txt.html#90228--90296,21,3.2,22,17) | 21 | 3.2 | 22 | 78 | 12 | 72 | 11 | 23 | 10 | 55 | 1.66 | *ycf2* (CDS) |
| 13 | [92682-92754](https://tandem.bu.edu/trf/output/tmpjfkfuxg0.2.7.7.80.10.50.500.1.txt.html#92682--92754,18,4.1,18,19) | 18 | 4.1 | 18 | 87 | 10 | 89 | 31 | 8 | 26 | 34 | 1.86 | *ycf2* (CDS) |
| 14 | [92700-92765](https://tandem.bu.edu/trf/output/tmpjfkfuxg0.2.7.7.80.10.50.500.1.txt.html#92700--92765,21,3.3,21,20) | 21 | 3.3 | 21 | 91 | 6 | 102 | 30 | 10 | 28 | 30 | 1.90 | *ycf2* (CDS) |
| 15 | [92701-92763](https://tandem.bu.edu/trf/output/tmpjfkfuxg0.2.7.7.80.10.50.500.1.txt.html#92701--92763,18,3.3,18,21) | 18 | 3.3 | 18 | 83 | 12 | 81 | 30 | 11 | 28 | 30 | 1.91 | *ycf2* (CDS) |
| 16 | [100497-100554](https://tandem.bu.edu/trf/output/tmpjfkfuxg0.2.7.7.80.10.50.500.1.txt.html#100497--100554,12,5.2,11,22) | 12 | 5.2 | 11 | 76 | 19 | 50 | 18 | 3 | 6 | 70 | 1.24 | *rps12/ trnV-GAC (IGS)* |
| 17 | [140755-140812](https://tandem.bu.edu/trf/output/tmpjfkfuxg0.2.7.7.80.10.50.500.1.txt.html#140755--140812,12,5.2,11,23) | 12 | 5.2 | 11 | 76 | 19 | 50 | 70 | 6 | 3 | 18 | 1.24 | *trnV-GAC/ rps12* (IGS) |
| 18 | [148546-148608](https://tandem.bu.edu/trf/output/tmpjfkfuxg0.2.7.7.80.10.50.500.1.txt.html#148546--148608,18,3.3,18,24) | 18 | 3.3 | 18 | 88 | 6 | 81 | 30 | 28 | 11 | 30 | 1.91 | *ycf2* (CDS) |
| 19 | [148544-148609](https://tandem.bu.edu/trf/output/tmpjfkfuxg0.2.7.7.80.10.50.500.1.txt.html#148544--148609,21,3.3,21,25) | 21 | 3.3 | 21 | 91 | 6 | 102 | 30 | 28 | 10 | 30 | 1.90 | *ycf2* (CDS) |
| 20 | [148555-148627](https://tandem.bu.edu/trf/output/tmpjfkfuxg0.2.7.7.80.10.50.500.1.txt.html#148555--148627,18,4.1,18,26) | 18 | 4.1 | 18 | 87 | 10 | 89 | 34 | 26 | 8 | 31 | 1.86 | *ycf2* (CDS) |
| 21 | [151014-151081](https://tandem.bu.edu/trf/output/tmpjfkfuxg0.2.7.7.80.10.50.500.1.txt.html#151014--151081,21,3.2,21,30) | 21 | 3.2 | 21 | 81 | 10 | 77 | 54 | 10 | 23 | 11 | 1.67 | *ycf2* (CDS) |

*IGS: if the start and end position is between two genes, CDS: is the start and end is within the CDS, Intron: if the start and end is within CDS but between two fragment of CDS.
